# The Transcription and Translation Landscapes during Human Cytomegalovirus Infection Reveal Novel Host-Pathogen Interactions

**DOI:** 10.1371/journal.ppat.1005288

**Published:** 2015-11-24

**Authors:** Osnat Tirosh, Yifat Cohen, Alina Shitrit, Odem Shani, Vu Thuy Khanh Le-Trilling, Mirko Trilling, Gilgi Friedlander, Marvin Tanenbaum, Noam Stern-Ginossar

**Affiliations:** 1 The Department of Molecular Genetics, Weizmann Institute of Science, Rehovot, Israel; 2 Institut für Virologie, Universitätsklinikum Essen, Universität Duisburg-Essen, Essen, Germany; 3 The Nancy & Stephen Grand Israel National Center for Personalized Medicine, Weizmann Institute of Science, Rehovot, Israel; 4 Department of Cellular and Molecular Pharmacology, University of California, San Francisco, San Francisco, California, United States of America; University of North Carolina at Chapel Hill, UNITED STATES

## Abstract

Viruses are by definition fully dependent on the cellular translation machinery, and develop diverse mechanisms to co-opt this machinery for their own benefit. Unlike many viruses, human cytomegalovirus (HCMV) does suppress the host translation machinery, and the extent to which translation machinery contributes to the overall pattern of viral replication and pathogenesis remains elusive. Here, we combine RNA sequencing and ribosomal profiling analyses to systematically address this question. By simultaneously examining the changes in transcription and translation along HCMV infection, we uncover extensive transcriptional control that dominates the response to infection, but also diverse and dynamic translational regulation for subsets of host genes. We were also able to show that, at late time points in infection, translation of viral mRNAs is higher than that of cellular mRNAs. Lastly, integration of our translation measurements with recent measurements of protein abundance enabled comprehensive identification of dozens of host proteins that are targeted for degradation during HCMV infection. Since targeted degradation indicates a strong biological importance, this approach should be applicable for discovering central host functions during viral infection. Our work provides a framework for studying the contribution of transcription, translation and degradation during infection with any virus.

## Author Summary

Viruses are fully dependent on the cellular translation machinery, and develop diverse mechanisms to co-opt it for their own benefit. However, fundamental questions such as: what is the effect that infection has on the spectrum of host mRNAs that are being translated, and whether, and to what extent, a virus possesses mechanisms to commandeer the translation machinery are still hard to address. Here we show that by simultaneously examining the changes in transcription and translation along Human cytomegalovirus (HCMV) infection, we can uncover extensive transcriptional regulation, but also diverse and dynamic translational control. We were also able to show that, at late time points in infection, translation of viral mRNAs is higher than that of cellular mRNAs. Lastly, we take advantage of our measurements of translation (protein synthesis rate) and integrate these with mass spectrometry measurements (protein abundance). This integration allowed us to unbiasedly reveal dozens of cellular proteins that are being degraded during HCMV infection. Since targeted degradation indicates a strong biological importance, this approach should be applicable for discovering central host functions during viral infection. Our work provides a framework for studying the contribution of transcription, translation and degradation during infection with any virus.

## Introduction

Human cytomegalovirus (HCMV) is a ubiquitous pathogen, infecting the majority of the human population worldwide, leading to severe diseases in newborns and immunocompromised adults. The HCMV genome contains almost 240kb, making it the largest known human virus. Its genome was traditionally estimated to code for approximately 200 open reading frames [1,2], but our recent study showed that many additional, mostly short open reading frames are also translated during infection [3].

During viral infection, cellular gene expression is subjected to rapid alterations induced by both viral and antiviral mechanisms. The differential regulation of cellular transcription and translation distinguishes host pathways that the virus either relies on or actively subverts and can open new therapeutic opportunities and reveal novel principles in cell biology. Over the years, a large body of work was conducted to decipher these changes in a global manner by examining the temporal changes in RNA levels [4–11]. The use of microarrays revealed many biological pathways that are significantly altered during infection and established an important progress in our understanding of how HCMV exploits cellular pathways during infection [6–11]. These studies helped to reveal numerous pathways that are elevated during infection and are important for viral propagation such as cell cycle, DNA damage, transcription and translation factors and energy production. In addition pathways that were reduced during infection were also mapped, such as cell adhesion, cytoskeletal regulators and apoptosis and extracellular matrix. Recently, advancement in mass-spectrometry methods had been used to quantify the cell proteome along HCMV infection giving a wider view on the pathways that are altered during infection [12]. More specifically, this method was used to predict natural killer (NK) and T cells ligands by identifying cell surface molecules which are downregulated during HCMV infection [12]. However, these approaches could not delineate transcriptional, translational and post-translational layers of regulation. It had been shown that unlike many other viruses (including several Herpes viruses, e.g. Herpes simplex virus 1 and 2), the overall impact of HCMV is stimulation of host RNA and protein synthesis [13–15]. Still, fundamental questions such as how and to what extent HCMV changes the spectrum of host mRNAs translation, and whether the virus possess mechanisms to ensure more effective translation of its own mRNAs, have just begun to be addressed [16,17]. In a recent study, changes in host genes translation at 48 hours post HCMV infection were examined by analyzing the fraction of mRNAs associated with polysomes. This study revealed that a significant fraction of cellular mRNAs are translationally activated or repressed by HCMV [17]. In addition, expression of pUL38, a virally encoded mTORC1-activator sufficed to partially recapitulate these translational alterations in uninfected cells, demonstrating that some of the effect is mediated by mTORC1 activation [17].

Here, we have used RNA sequencing (RNA-seq) and ribosome profiling (deep sequencing of ribosomes-protected fragments) to globally map the changes in host genes transcription and translation during HCMV replication. These comprehensive and simultaneous measurements revealed how HCMV orchestrates cellular gene expression at both the transcription and translation levels. We identified several novel pathways that are upregulated during infection and are central for viral propagation. We show that most of the regulation of cellular genes along infection occurs on the level of transcription but our experiments also uncover extensive and dynamic translational regulation of subsets of cellular genes. In addition, our measurements enabled the comparison between translation of viral and host genes, revealing that late in infection viral genes are translated more efficiently than their host counterparts. Finally, by integrating our measurements of protein synthesis rate with recent measurements of protein levels [12] we were able to globally and unbiasedly identify host proteins that are actively targeted for degradation during HCMV infection. We show that BTN2A1 and IGSF8, two cell surface proteins that belong to the immunoglobulin (Ig) superfamily are degraded during HCMV infection. We also reveal that few cytosolic proteins including ROCK1, a key regulator of actin cytoskeleton, are degraded during HCMV infection.

## Results

### Simultaneous monitoring of RNA levels and translation during HCMV infection

**Fig 1:**
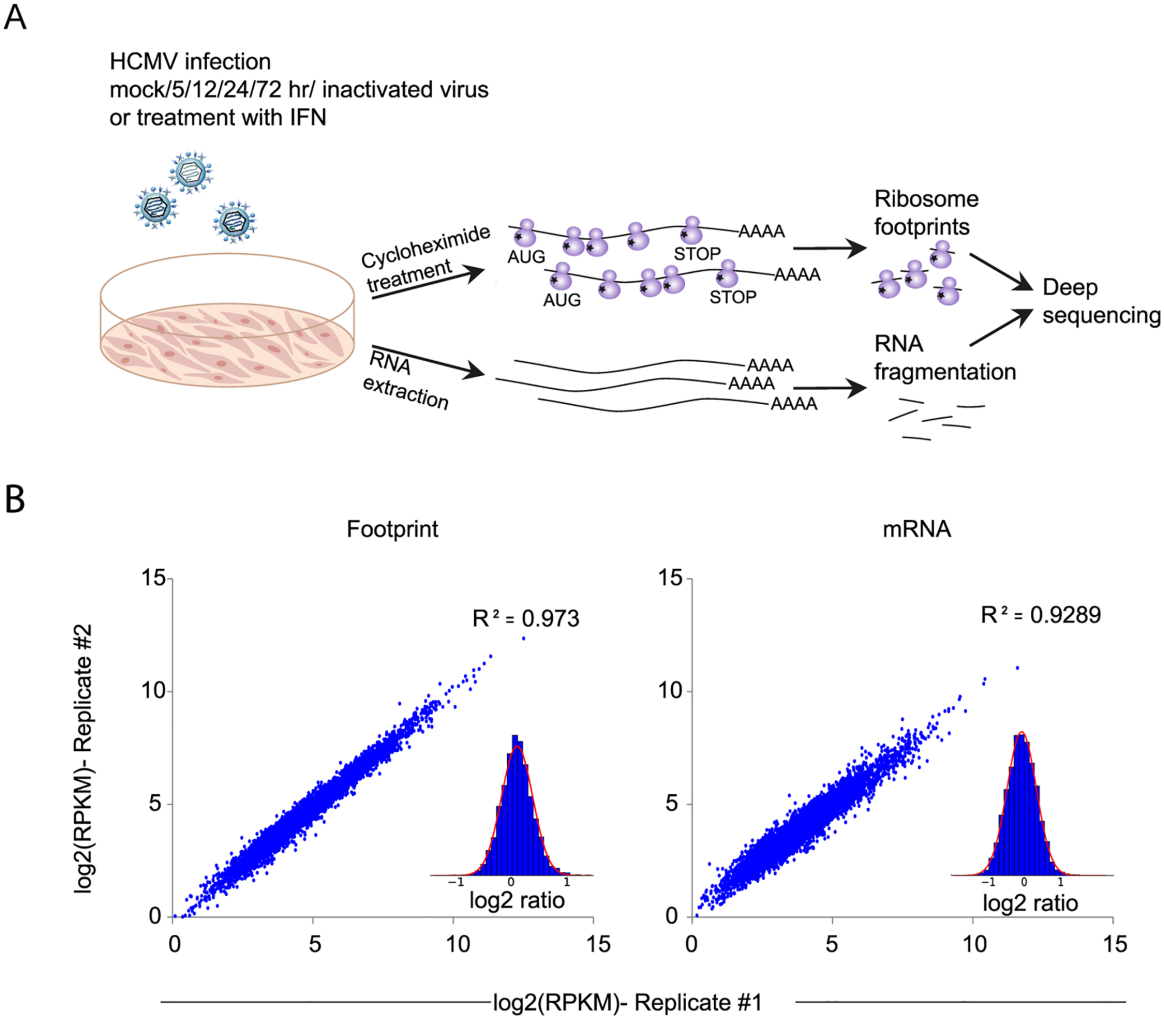
Experimental approach for mapping mRNA abundance and protein production rate through the course of HCMV infection. **A.** Primary fibroblasts were infected with HCMV and harvested at different times after infection for ribosome footprints and RNA-seq analysis. **B.** Reproducibility of the ribosome occupancies and mRNA measurements of host genes at 72hpi. The correlations in footprints and mRNA measurements between biological replicates are presented.

**Fig 2:**
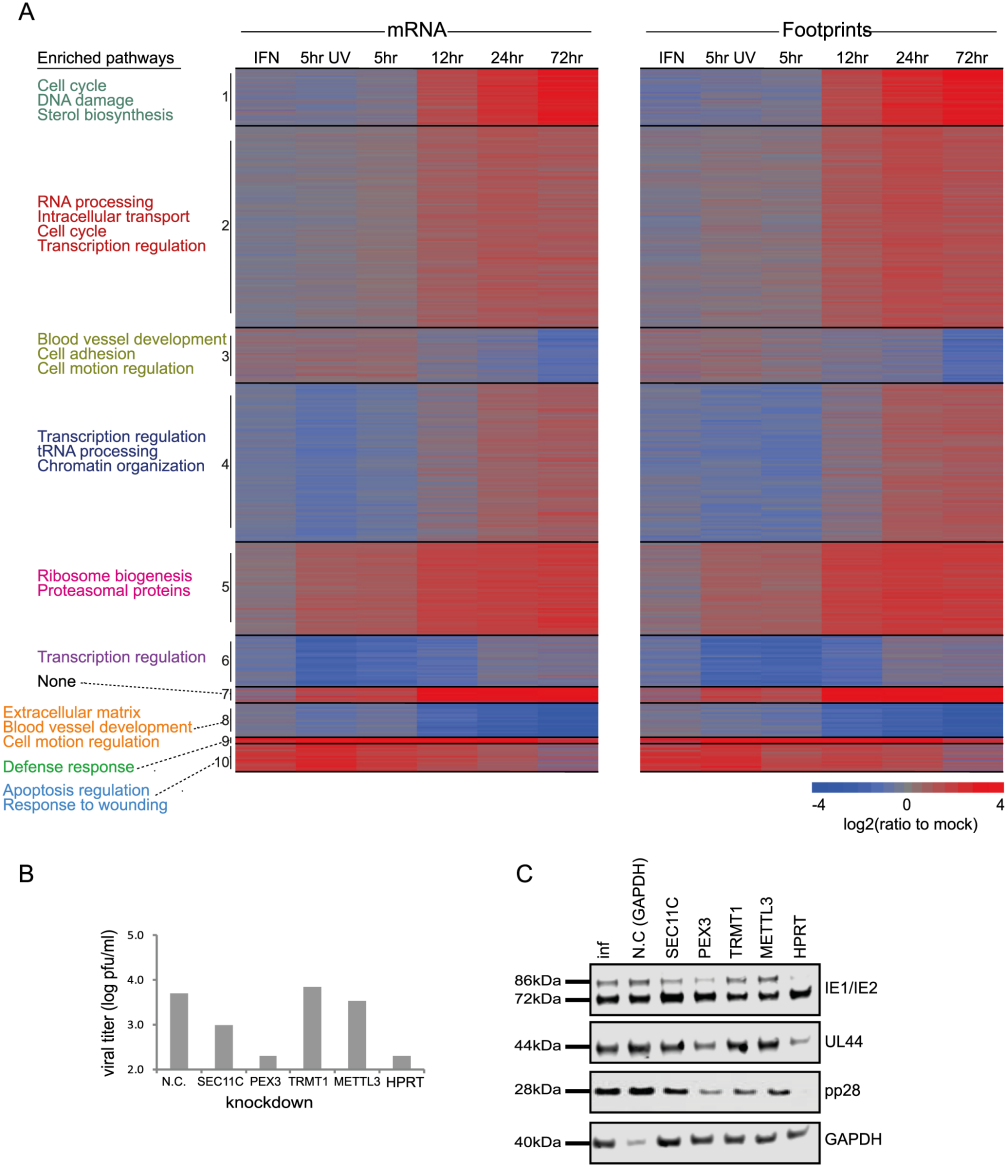
Changes in cellular gene expression during HCMV infection. **A.** Ribosome footprints and mRNA read densities (reads per kilobase million, RPKM) of well-expressed human transcripts after treatment with interferon (IFN), infection with inactivated virus (5hr UV) and across four time points during HCMV infection were calculated relative to expression in uninfected cells (mock). Shown is heat map of log_2_ expression ratios after partitioning clustering. The ten main clusters are marked and for each of these clusters the pathway enrichment (Benjamini < 1E-5) is labeled on the left. **B.** Cells transfected with a control or siRNAs targeting different host genes were infected with the Merlin strain (MOI = 3). After 5 days supernatants were collected and viral titers were calculated by TCID50. Each experiment was performed in triplicates and results shown are representative. **C.** Samples from experiments detailed in (B) were analyzed by western blot for viral proteins expression from immediate early, early and late stages of infection (IE1/IE2, UL44 and pp28, respectively).

To gain a detailed view of the changes that occur in host genes transcription and translation over the course of HCMV infection, we infected human foreskin fibroblasts (HFF) with the Merlin HCMV strain and harvested cells at 5, 12, 24 and 72 hours post infection (hpi). We also used cells treated with type I interferon (IFN) or cells infected with an irradiated virus, in which viral DNA is inactivated [18], preventing viral gene transcription. We designed our experiment to simultaneously monitor both RNA levels and translation (Fig 1A). Deep sequencing of mRNA (RNA-seq) allows a detailed mapping of transcript levels during infection and these were paired with ribosome footprints (deep sequencing of ribosome-protected mRNA fragments), which allow accurate measurement of protein synthesis by capturing the overall *in vivo* distribution of ribosomes on a given message [19]. In order to assess the reproducibility of our experiments we have prepared two independent biological replicates for the 5hpi and 72hpi time points. Both the mRNA and footprints read density measurements were highly reproducible (correlation coefficient [R^2^] = 0.97 and 0.92; SD in ratio between biological replicates corresponded to a 0.18- and 0.3-fold change, respectively) demonstrating consistency in our experimental methods (Figs 1B and S1). We quantitatively assessed the expression pattern of 10,354 genes. Interestingly, 73% of the transcripts changed by more than 3-fold in their footprints densities along infection, reflecting the drastic changes that occur in cells during HCMV infection (S1 Table). In order to identify patterns of specific cellular pathways that were influenced during infection, we compared the expression of infected samples to mock sample and clustered the mRNA and footprints ratios using partitioning clustering. This approach allowed clustering of the cellular transcripts into ten distinct classes based on similarities in temporal expression profiles in the RNA-seq and ribosome profiling data. Overall we found that changes in ribosome footprints tracked the changes in transcripts abundance (Figs 2A and S2A), which indicates that most of the regulation of host gene expression occurs at the level of transcription. To identify biological processes that are altered during HCMV infection we applied the DAVID software [20]. Most of the time-related clusters showed enrichment for specific biological functions supporting their biological relevance (Fig 2A). Many of these biological functions were captured by previous transcriptomic studies [4,14,15,8,9] and were reported to be affected by HCMV infection such as cell cycle, DNA damage, sterol biosynthesis, ribosome biogenesis and the proteasome [21–25]. The increased sensitivity of deep sequencing approaches allowed us to also identify significant changes in novel biological functions such as RNA processing, protein intracellular transport and transcription regulation. The temporal profile of candidate genes from each of these clusters was confirmed by real-time PCR or western blot analysis (S2B Fig). The full DAVID enrichment analysis is presented in S2 Table.

### Identification of up- or down-regulated pathways during HCMV infection

It is likely that many of the genes in the same temporal profile are co-regulated by common transcriptional activators or repressors. Therefore, to find potential regulators that drive the expression of these clusters and to better focus on well-characterized canonical pathways, we turned to Ingenuity Pathway Analysis (IPA) (Ingenuity Systems, www.ingenuity.com). This analysis helped to reveal functional pathways that are significantly changing during infection and identifying upstream molecules that may control the expression of the genes in each cluster.

HCMV is known to induce the cellular DNA damage response (DDR), that includes activation of the ATM, H2AX, NBS1, CHK2, CHK1 and p53 genes [21]. Indeed, we found multiple pathways related to DNA damage that were significantly enriched in cluster 1 (S3 Table). Interestingly, we identified significant upregulation of genes that promote homologous recombination repair (HR), such as Fanconi anemia complementation group D2 protein (FANCD2), BRCA1 and BARD1 (S3 Fig, P-val = 1.25E-16). Recently, it had been suggested that FANCD2 upregulation during HSV1 infection is important for promoting HR on the expanse of non- homologous end-joining [26]. Our results, therefore, suggest that similar strategy may be used in HCMV-infected cells. We also identified significant upregulation of DNA mismatch repair complex that is required for the repair of DNA replication errors (S4 Fig, P-val = 6.3E-12). Mismatch Repair Proteins were shown to be elevated and required for efficient HSV1 replication [27]. The significant elevation we mapped during HCMV infection suggests that the mismatch repair may also be important during HCMV infection and it will be of interest to determine whether mismatch repair proteins contribute to the fidelity of herpesvirus DNA replication. An important upstream regulator of genes in cluster 1 was E2F1 (P-val = 3.14E-16) which was previously shown to be important for HCMV replication [28].

Cholesterol levels are significantly elevated during HCMV infection and cholesterol import was shown to play an important role in this process [23]. Our results also reveal direct upregulation of cholesterol biosynthesis enzymes implying an increase in cholesterol *de novo* synthesis (P-val = 1.7E-6, cluster1, S3 Table).

In agreement with the overall upregulation in translation in HCMV infected cells [17], we found translation initiation factors to be significantly elevated (S5 Fig, P-val = 7.94E-11). Cluster 2 is also enriched in genes related to assembly of RNA polymerase II (S6 Fig, P-val = 2.4E-5), suggesting a simultaneous upregulation of both the transcription and translation machineries. In addition, we identified upregulation of genes related to tRNA charging and tRNA modifications (P-val = 3.3E10-5, cluster 4, S3 Table) that probably support the higher demand for tRNAs due to the increase in translation.

We have also mapped a significant enrichment in genes involved in proteins quality control (P-val = 5.03E-13, S7 Fig), including upregulation of many of the cell’s chaperones, the proteasome components and deubiquitinating enzymes (DUBs). DUBs form a large family of proteases that cleave ubiquitin chains from target proteins and their up regulation can effect the stability, localization and function of the proteome [29]. Interestingly, DUBs are also encoded by herpesviruses and HCMV encoded DUB (UL48) was shown to influence viral replication [30]. It is possible that the increased cellular DUB activity can increase the stability of target proteins during the progression of infection by inhibiting polyubiquitination.

NRF2 is a transcription factor that plays a key role in cellular defense against oxidative stress. We observed upregulation in NRF2 targets, that included the induction of few detoxifying enzymes and stress response proteins (P-val = 6.6E-5, cluster 5, S3 Table). These results extend previous observations showing NRF2 was elevated during HCMV infection[31].

Downregulated genes (Clusters 3 and 8) were significantly enriched (P-values 1.4E-8 and 1.25E-14 respectively) for genes related to the synthesis of the extra cellular matrix (ECM) (S8 Fig) and for metalloproteinase inhibition (P-values 1.2E-3 and 8.3E-6, S9 Fig), in agreement with previous microarray measurements [9]. Interestingly, collagen was found to restrict HSV-1 infectivity in healthy tissues [32], suggesting a potential motivation to downregulate ECM production. Reduction in metalloproteinase inhibition may have additional effect on reducing ECM protein levels but can also affect proteolytic degradation of cell surface molecules. Indeed, increased proteolytic degradation was demonstrated for the cell surface associated low density lipoprotein related receptor 1 (LRP1) at late phase of infection[23]. In addition proteolytic degradation of cell surface molecules could be employed by the virus to increase shedding of immune stimulatory molecules [33]. Important upstream regulators of genes in these downregulated clusters are β-catenin (Pval = 3.96E-11) and TP63 (Pval = 3.14E-11), in agreement with recent studies showing dysregulation of Wnt/β-catenin signaling pathway during HCMV infection[34]. The enrichment of all pathways and upstream regulators in the different clusters are presented in S3 Table and S4 Table.

In order to test the functional importance of genes we identified as elevated during infection, we tested the effect of knockdown (KD) of few candidate genes on viral replication. We chose genes that fall in different functional categories that were not connected before to HCMV infection. Since we observed elevation in genes responsible for protein transport and localization we targeted two genes that are involved in these processes; SEC11C- a component of the microsomal signal peptidase complex that removes signal peptides from nascent proteins and PEX3- which is involved in peroxisome biosynthesis and integrity; We also chose two genes that are involved in RNA processing; TRMT1-an enzyme that dimethylates a guanine residue on most tRNAs but its importance is poorly defined and METTL3- N6-methyltransferase that methylates adenosine residues in mRNAs and was recently shown to be important for RNA stability and translation [35]. Lastly we chose HPRT1- which is a central enzyme in the purine salvage pathway. Since the expression of these genes could be essential for cell survival, we confirmed that the various KDs did not cause significant cell death and that siRNA-mediated ablation reestablished mRNA levels to these observed in uninfected cells (S10A and S10B Fig). Importantly, KD of SEC11C, PEX3 and HPRT1 significantly reduced viral titers (Fig 2B). In order to preclude off target effects we confirmed that similar effects were obtained using distinct siRNAs that target the same gene (S10C Fig). These results strongly indicate that the elevation in the secretory pathway proteins, the integrity of peroxisomes and the purine salvage pathway are all contributing to viral propagation. Importantly, both PEX3 and HPRT1 KD blocked HCMV at early stages of infection as the expression of early-proteins IE2 and UL44 was reduced (Figs 2C and S10D). In contrast, SEC11C blocked only the late stage of infection as only the expression of the late-protein, pp28 was reduced (Figs 2C and S10D). Although the TRMT1 KD did not significantly affect viral titers we observed a reproducible reduction in pp28 expression (Figs 2C and S10D) suggesting that the TRMT1 dimethylation activity might be playing a subtler role during HCMV infection. Overall, our results increase the breadth of knowledge about the changes that occur in various cellular processes during HCMV infection.

### Evaluating the role of translation regulation during HCMV infection

**Fig 3:**
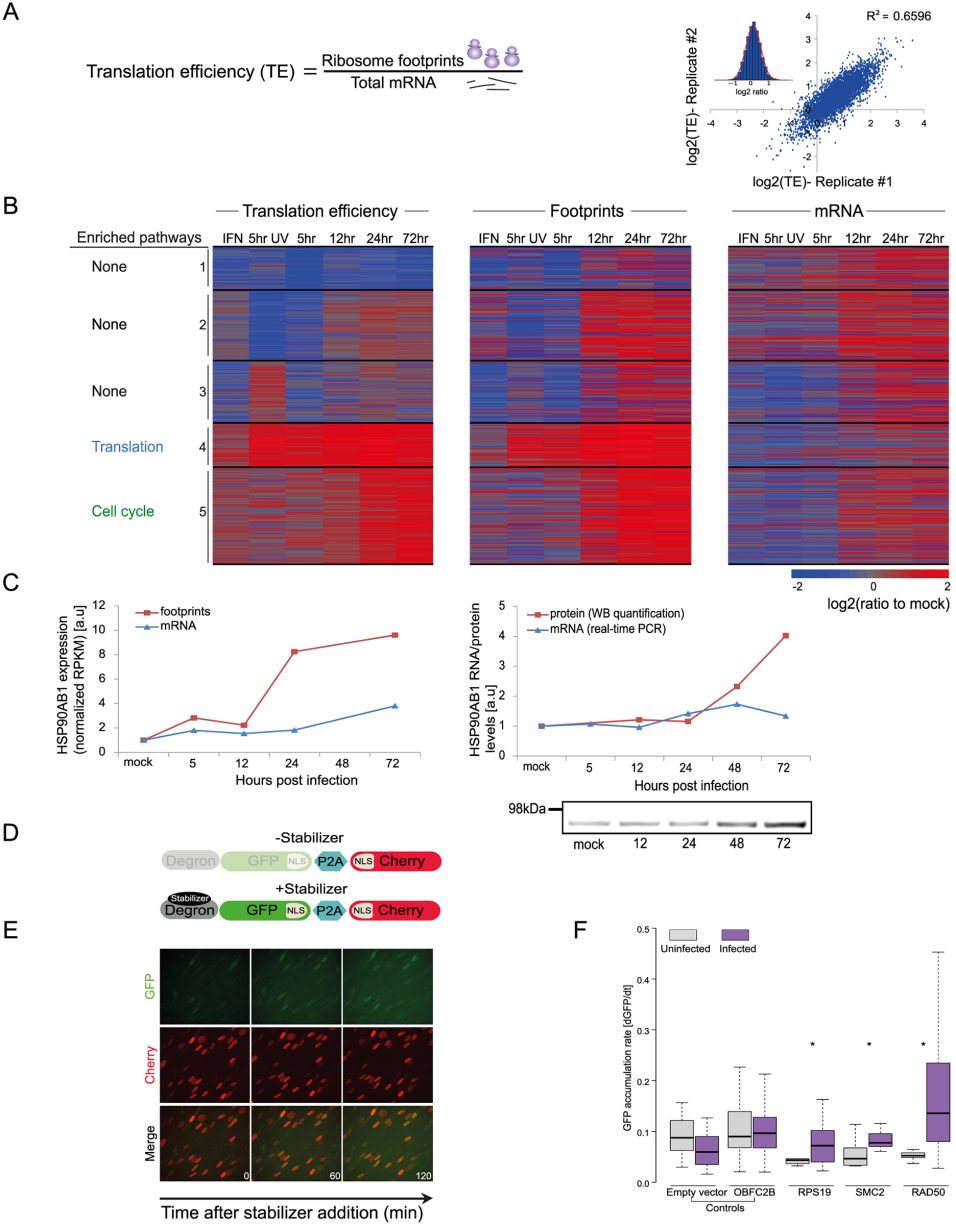
Dynamic changes in translation efficiency of cellular genes during HCMV infection. **A.** TE was calculated by dividing RPKM of ribosome footprints with RPKM of mRNA measurements and the correlation in TE of host genes between biological replicates is represented. **B.** TE of 731 human transcripts after interferon (IFN) treatment, infection with inactivated virus (5hr UV) and during HCMV infection were calculated relative to TE in uninfected cells. The TE ratios were subjected to partitioning clustering and shown is the heat map of log_2_ TE ratios and the corresponding footprints and mRNAs ratios. The five clusters are marked and for each of these clusters the pathway enrichment (P-val < 1E-4) is labeled on the left. **C.** HSP90AB1 mRNA and footprints levels as measured in our RNA-seq and ribosome profiling experiment (left panel) and verification of these measurements by real-time PCR and western blot analysis (right panel). Real-Time PCR data was normalized by the amount of *polr2l* mRNA. **D.** Schematic representation of the live cell translation reporter. An inducible degron (DHFR-Y100I) fused to sfGFP-NLS is separated from an NLS-mCherry protein by a P2A ribosome skipping sequence, which allows these two proteins to be synthesized separately from a single transcript. In the absence of the stabilizer, trimethoprim (TMP), sfGFP-NLS is degraded, while NLS-mCherry is stable. Upon addition of TMP, DHFR-sfGFP-NLS is stabilized and GFP fluorescence increases over time due to GFP protein synthesis, thus providing readout for TE. **E.** HFF cells stably expressing the reporter were treated with 3 μM TMP and then imaged every 60 min. As can be seen in representative images the GFP intensity increases over time, while the mCherry signal remains constant. **F.** 5’ UTRs of indicated genes were cloned upstream of the GFP, and HFF cells stably expressing these constructs were created. To measure translation cells were either infected with HCMV for 24hr or left uninfected, then TMP was added to the infected and uninfected cells and the cells were imaged for 3hr. The increase in GFP fluorescence was measured and normalized (see Methods). Shown boxplot represent the GFP accumulation, cells expressing the 5’UTRs of RPS19, SMC2 and RAD50 showed significant higher accumulation of GFP in infected cells compared to uninfected (* P-val < 0.05).

To quantitatively evaluate the role of translational control along HCMV infection, we calculated relative translation efficiency (TE) across our time course. TE is defined as the ratio of ribosome-associated RNA (footprints) to total mRNAs for a given gene (Fig 3A). Replicates indicated high reproducibility (SD of ratio between biological replicates corresponded to a 0.29 fold change), which allowed sensitive measurement of dynamic translational control (Figs 3A and S11A). In order to focus on cases in which translational regulation might play a substantial role in viral replication, we centered on genes that showed more than a 3-fold difference in their TE between any two-time points. In addition, we required that the change in TE would be accompanied by a change in a similar direction in the footprints densities. Based on these criteria we obtained 731 transcripts that showed significant changes in their TE during infection (S5 Table). For each of these genes we calculated log_2_ TE versus the mock sample and clustered them into five classes based on similarity in their temporal TE profiles. The heat-map of the footprints and mRNA temporal profiles of these genes exemplifies the translation regulation; the changes in footprints are much more pronounced than the changes in mRNA levels (Fig 3B). To characterize the parameters involved in HCMV-dependent translational regulation, we examined specific characteristics of the 5’ untranslated regions (5’UTRs) of the corresponding transcripts, including their length and percent of G+C content. We observed few unique features that define genes from each of these clusters (S11B and S11C Fig), suggesting that several features of 5’UTR may contribute to the TE along infection. In addition, we examined whether the TE clusters show strong enrichment for specific biological processes. Interestingly, cluster 4 (in which translation is induced at 5hpi) is enriched in genes related to the translation machinery, including many of the ribosomal proteins and translation initiation factors. This enrichment suggested that elevated translation of these genes is mediated by mTORC1 activation, as many of mTORC1 targets are related to protein synthesis [36,37]. Supporting this notion, there is the significant overlap (Pval = 5.432e-21) between mRNAs whose translation is repressed by mTOR inhibitors [38,39] and genes found in cluster 4. Genes in cluster 5 (in which translation is induced at 24hpi) were significantly enriched in functions related to cell cycle regulation. Since HCMV is known to arrest the cell cycle at a “pre-S” phase we speculated that translation regulation of this cluster could be attributed to the cell cycle arrest. Indeed, mRNAs whose translation was upregulated in this cluster significantly overlapped with genes that showed enhanced translation at G1 and S phases of the cell cycle (Pval = 9.03e-07 and Pval = 0.0007, respectively) [40]. Further clusters showed interesting patterns of translation regulation but were only weakly enriched for particular biological process (S6 Table). We confirmed the translational regulation for HSP90AB1, one of the genes identified in cluster 5, by showing that protein amounts increase throughout infection while no significant changes in mRNA as measured by real-time PCR are observed (Fig 3C). In order to further validate our translation measurements and to generate a platform that will enable identification of *cis*-regulatory elements that control translation during HCMV infection, we used a fluorescence-based reporter system to analyze translation of individual transcripts in single, living cells (Figs 3D, 3E, and S11D and [41,42]). The 5’UTRs of translationally regulated genes from clusters 4 and 5 were inserted into the fluorescent reporter and compared with 5’ UTRs of controls mRNAs that did not show HCMV related translational regulation. While the 5’ UTRs of control transcripts did not show significant changes in translation after HCMV infection, the transcripts with 5’UTRs from translationally regulated genes (RPS19, SMC2 and RAD50) showed increased translation after HCMV infection (Fig 3F). These results provide validation of our TE calculations and demonstrate that the regulatory elements required for translational activation of these genes are present in their 5’UTRs. Overall, our data demonstrates that multifaceted translational control of gene expression is carried out during HCMV infection.

### Translation efficiency of viral genes outcompetes cellular genes late in HCMV infection

**Fig 4:**
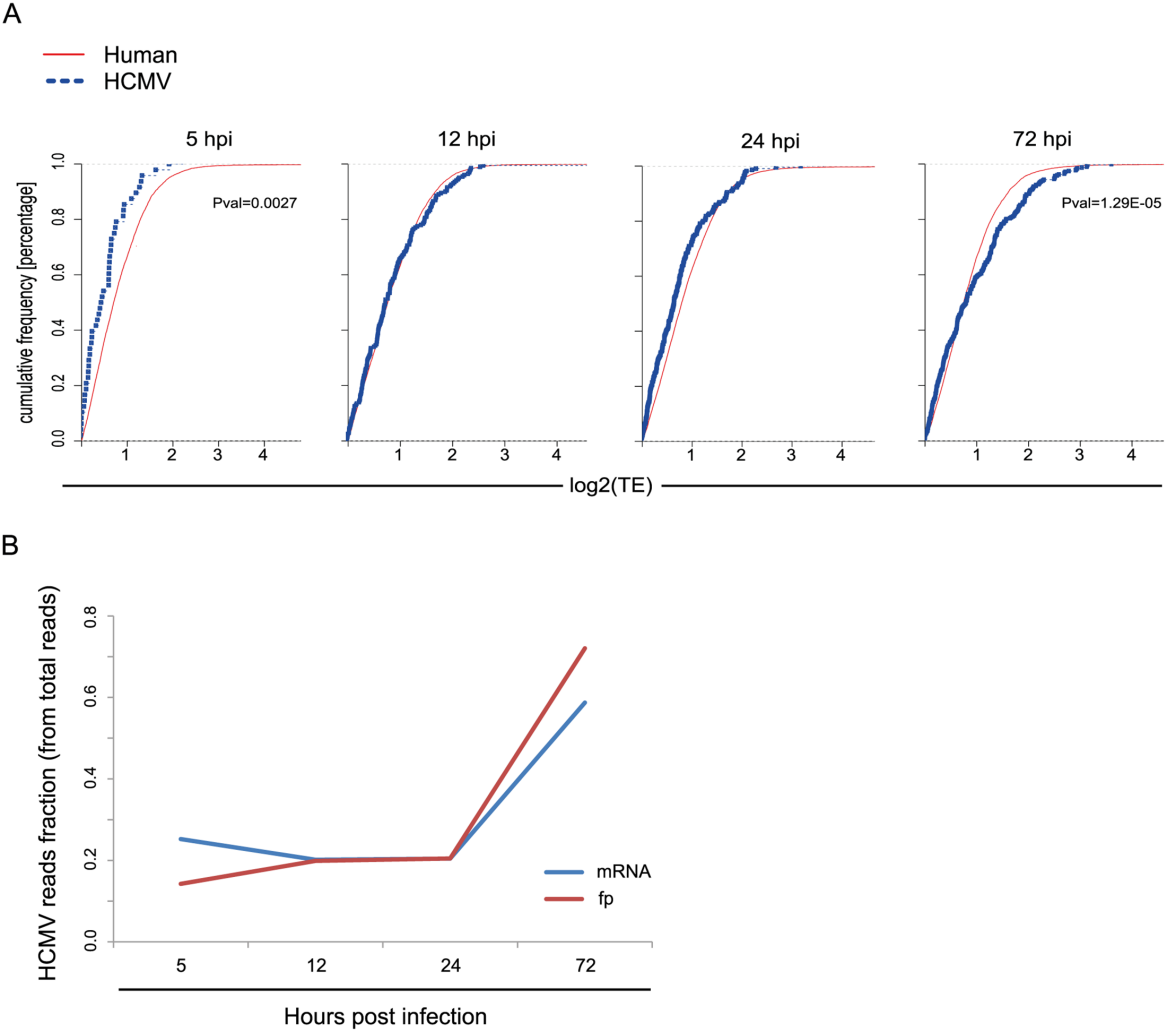
Differences in translation efficiency between viral and human genes along HCMV infection. **A.** Cumulative TE distribution among well-expressed human and viral genes shows that viral genes are translated less efficiently than cellular genes at 5hpi but more efficiently at 72hpi. P-values were calculated by Kolmogorov Smirnov test. **B.** The percentage of mRNA and footprints reads that maps to the virus are plotted along infection.

Replication of viruses is completely dependent on the host translational apparatus and many viruses commandeer this machinery to translate their own mRNAs on the expense of cellular mRNAs. In order to evaluate if HCMV evolved mechanisms to co-opt the cells' ribosomes we compared the TE of human genes to that of viral genes at each of the time points along infection. Interestingly, at 5hpi, on average, viral genes are translated less efficiently than human genes (Fig 4A, Pval = 0.0027), this suggests a successful host defense mechanism that lessens the translation of viral mRNAs compared to host mRNAs at the beginning of HCMV infection. However, this effect is diminished later during infection, as at 12hpi and 24hpi human and viral genes do not show any significant difference in their TE. Interestingly, at 72hpi viral mRNAs are, on average, translated more efficiently than human mRNAs (Fig 4A, Pval = 1.29E-05). These same effects were observed in independent biological replicates (S12 Fig). Thus, translation rates of viral mRNAs late in infection are higher than expected from their mRNA prevalence. This effect can also be seen when the fraction of mRNA and footprints reads that map to the virus are plotted along infection (Fig 4B). The molecular mechanism underlying this phenomenon is yet to be studied.

### Uncovering immune ligands that are degraded during HCMV infection

**Fig 5:**
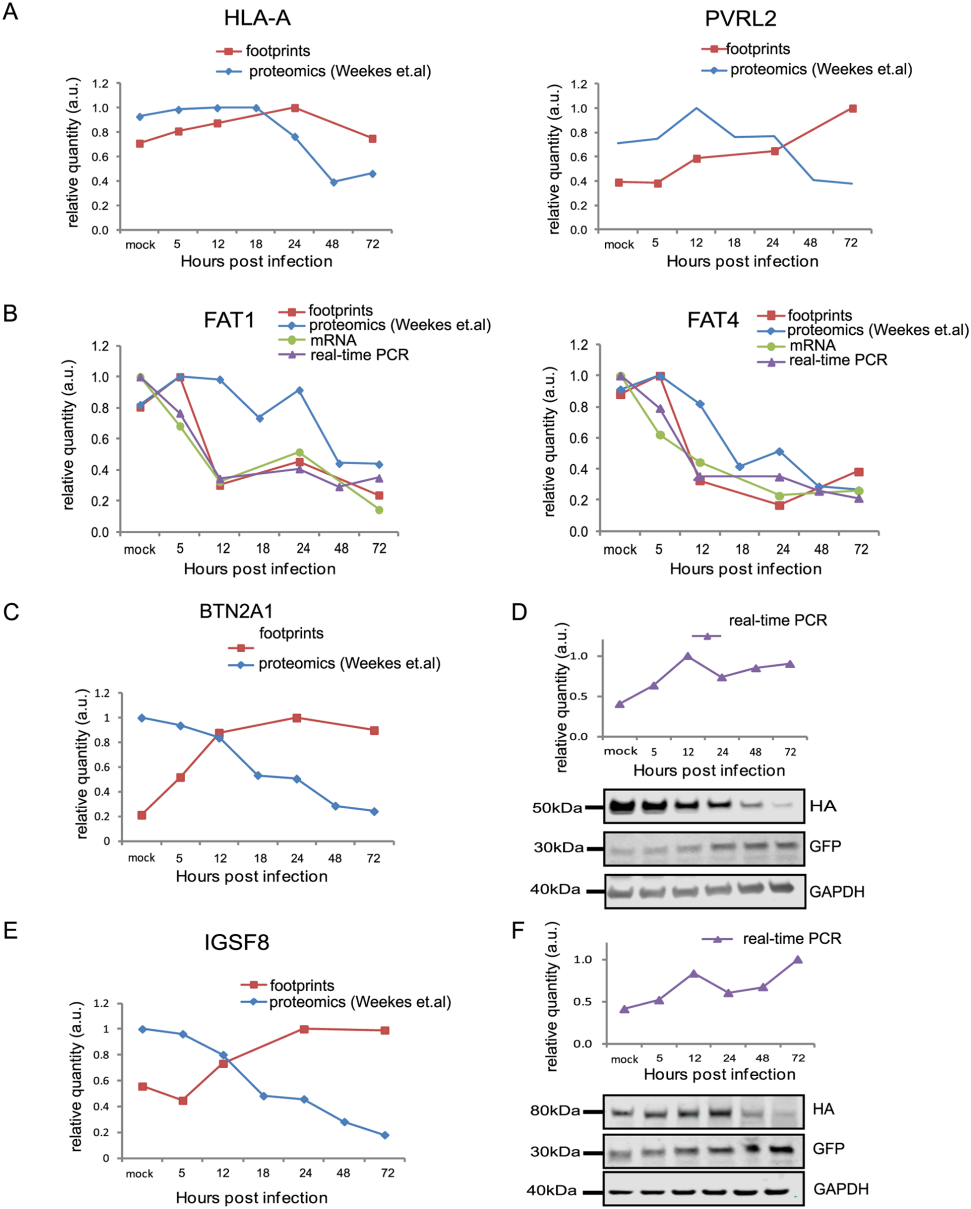
Integration between ribosome footprints and protein abundance allows detection of immune ligands that are degraded during infection. **A.** Ribosome profiling measurements for HLA-A and the NK ligand PVRL2 compared with temporal protein expression measured [12]. **B.** Expression of FAT1 and FAT4 measured by ribosome profiling, RNA-seq and real-time PCR analysis compared with protein abundance [12]. **C and E.** Ribosome profiling measurements of BTN2A1 and IGSF8 compared with protein abundance [12], respectively. **D and F.** Real-time PCR analysis of *btn2a1* (D) and *igsf8* (F) mRNA levels along HCMV infection (upper panels). HFF cells stably expressing BTN2A1-HA or IGSF8-HA were infected with HCMV. Protein levels were detected by western blot analysis using anti-HA antibody (lower panels). GFP levels (expressed from the same vector) were used as internal control. Real-time PCR data was normalized by the amount of *mfge8* mRNA. Each experiment was performed in triplicates.

**Fig 6:**
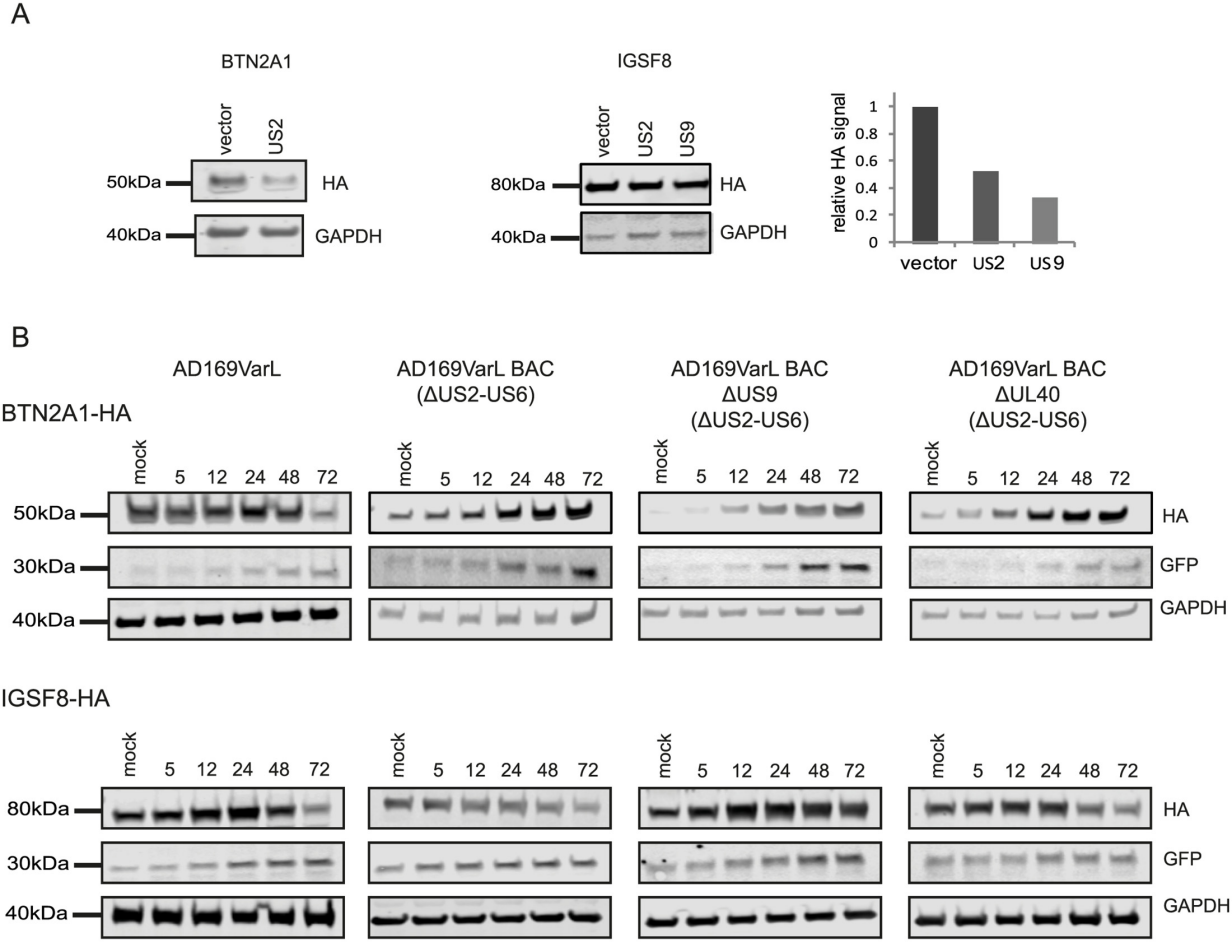
Identification of viral proteins responsible for BTN2A1 and IGSF8 degradation. **A.** HFF cells stably expressing BTN2A1-HA or IGSF8-HA were transduced with lentiviral vector expressing either US2, US9 or empty vector. BTN2A1 and IGSF8 protein levels were measured by immunoblotting. Right panel represents the quantification of IGSF8 expression relative to GAPDH amount from the same cells. **B.** HFF cells stably expressing BTN2A1-HA or IGSF8-HA were mock-infected or infected with HCMV AD169VarL, AD169VarL-BAC, AD169VarL-BACdeltaUS9 or AD169VarL-BAC deltaUL40 in MOI = 5 for 72 hr. BTN2A1 or IGSF8 protein levels were analyzed along infection by immunoblotting detection of the HA signal.

HCMV is a paradigm for viral immune evasion and several immune ligands were shown to be targeted for proteosomal degradation by specific viral proteins [43]. One prediction is that molecules that play a role in marking infected cells for immune recognition will be induced at the transcriptional and translational levels (rate of protein synthesis) as a cellular antiviral response, while the virus will stimulate their degradation, causing their protein levels to drop. A recently published quantitative proteomic analysis of host proteins levels during HCMV infection [12] identified many immune molecules that are down regulated during infection. However, down regulation of a given protein could be merely a mirror of transcriptional changes and therefore, does not necessarily indicate degradation. We reasoned that comparing our footprints measurements with these quantitative proteomic measurements, would allow us to systematically identify immune molecules that are targeted for degradation. Indeed, when we examined the profiles of proteins that were shown to be targeted for degradation by HCMV, such as; HLA-A (targeted by US2 and US11 proteins [44,45]) and PVRL2 (a ligand for the activating NK receptor DNAM-1 which is targeted by UL141 [46]), we observed the expected profiles (Fig 5A). We next examined the temporal profiles of the known NK and T cells activating or co-stimulatory ligands for which we had quantitative measurements, including protocadherins that were recently suggested to act as novel activating ligands for NK cells [12]. However, unlike HLA-A and PVRL2 we observed that the regulation of these immune ligands occurs mostly at the transcriptional level as both footprints and mRNA levels were downregulated along infection (Figs 5B and S13). Since active degradation during HCMV infection may indicate biological importance, we looked for inverse correlation between the footprints and protein temporal profiles in a list of potential immune ligands composed of proteins that belong to a few immune-related protein families [12]. This search led us to identify two proteins, BTN2A1 and IGSF8, which belong to the immunoglobulin superfamily and present a profile that suggests degradation (Fig 5C and 5E). Interestingly a recent study that performed plasma membrane profiling showed that the relative abundance of both BTN2A1 and IGSF8 was higher in cells infected with HCMV ΔUS2 compared to those infected with WT virus, further supporting the possibility that these proteins are actively degraded [47]. Real-time PCR measurements from cell lysates along HCMV infection supported elevation in BTN2A1 and IGSF8 mRNA levels (Fig 5D and 5F, upper panels). Since we were not able to obtain specific detection of BTN2A1 and IGSF8 using commercially available antibodies, we expressed these proteins fused to an HA tag in fibroblasts using lentiviral vectors. The vectors also contained green fluorescent protein (GFP) that was expressed from the same transcript using an internal ribosome entry site (IRES) and was used as an internal control. These cells were then infected with HCMV and the kinetics of BTN2A1 and IGSF8 expression along infection was evaluated by western blot analysis. These experiments demonstrated that BTN2A1 and IGSF8 are targeted for degradation since their protein levels were downregulated during HCMV infection (Fig 5D and 5F, middle panels), whereas the levels of the GFP which was expressed from the same transcript was elevated (Fig 5D and 5F, lower panels). Since these proteins were suggested to be effected by the viral US2 protein [47], we tested if US2 affect BTN2A1 and IGSF8 expression by ectopically expressing US2 in fibroblasts expressing either tagged BTN2A1 or tagged IGSF8. Importantly, US2 ectopic expression was sufficient to downregulate BTN2A1 and to lesser extent IGSF8 in uninfected fibroblasts (Fig 6A). We next tested the expression of BTN2A1 and IGSF8 during infection with the AD169VarL virus and a BAC derived AD169VarL virus, in which the US2-US6 region was deleted [48] (the region in which the BAC cassette was inserted). Similar to the results obtained with Merlin strain, both BTN2A1 and IGSF8 were degraded when cells were infected with the AD169VarL parental virus (Figs 6B, S14A, and S14B). In accordance with the plasma profiling results [47], in cells that were infected with the AD169VarL-BAC virus (that is US2-deleted) the expression of BTN2A1 was elevated and resembled the expression pattern of the GFP that was expressed from the same plasmid, strongly suggesting that US2 is essential and sufficient for BTN2A1 degradation (Figs 6B and S14A). Interestingly, however, although in the absence of US2 some of IGSF8 expression was restored, IGSF8 levels are still reduced during infection with the AD169VarL-BAC virus (Figs 6B (second panel) and S14B), indicating that additional viral proteins mediate IGSF8 degradation. In order to identify the viral protein/s that are involved in IGSF8 degradation we performed a pull-down on cells expressing HA tagged-IGSF8 and that were infected with HCMV for 48hr. Isolated viral and host proteins were resolved by electrophoresis and identified by mass spectrometry. Two HCMV proteins were identified in this capture experiment. The first was US9, which was recently shown to selectively target MICA*008 to proteasomal degradation [49] and the second was UL40, which possesses a signal peptide that mimics cellular signal peptides from HLA molecules and regulates the cell surface expression of the NK cell ligand HLA-E [50]. In order to test if these proteins are involved in IGSF8 degradation, we tested the expression of IGSF8 during infection with AD169VarL-BAC derived virus in which US9 or UL40 were deleted. In cells that were infected with the AD169VarL-BAC delta US9 virus, the expression of IGSF8 was elevated and better resembled the GFP levels that was expressed from the same plasmid, suggesting that US9 is contributing to IGSF8 degradation (Figs 6B and S14B). The effect of US9 seemed specific for IGSF8 since US9 deletion had no additional effect on BTN2A1 expression (Figs 6B and S14A, and S14B). In contrast to US9, the deletion of UL40 had no obvious effect on BTN2A1 or IGSF8 expression (Figs 6B, fourth panel, S14A, and S14B). Finally we tested if US9 affect IGSF8 by ectopically expressing US9 in fibroblasts expressing epitope tagged IGSF8. In accordance with the results obtained with US2, ectopic expression of US9 had mild but reproducible effect on IGSF8 expression in uninfected fibroblasts (Fig 6A, middle and right panel). Overall, these results illustrate how our ribosome profiling data in conjunction with recent mass spectrometry measurements [12] allowed us to identify two Ig superfamily proteins that are degraded during HCMV infection.

### Uncovering novel host proteins that are degraded during HCMV infection

**Fig 7:**
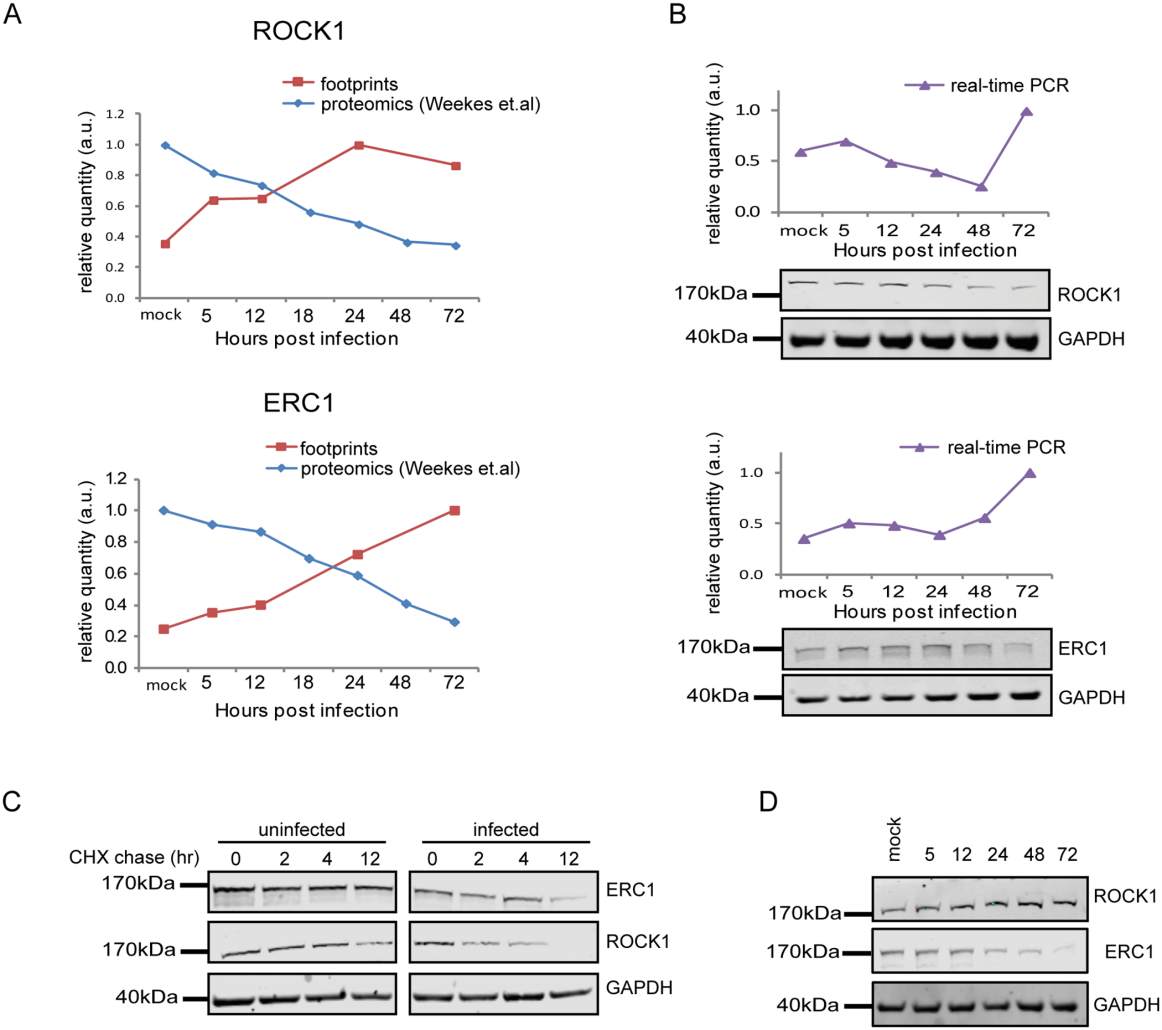
Integration between ribosome footprints and protein abundance measurements allows detection of cellular proteins that are degraded during HCMV infection. **A.** Ribosome profiling measurements of ROCK1 and ERC1 compared with protein abundance [12].**B.** Real-time PCR analysis of *rock1* and *erc1* with parallel measurements of protein levels by western blot analysis. Real-time PCR data was normalized by the amount of *mfge8* mRNA. Each experiment was performed in triplicates. Western blot analysis was performed on cell lysates and GAPDH was used as loading control. **C.** Cells were mock-infected or infected with HCMV for 48hr and cycloheximide (25μg/ml) was added to the medium to stop protein translation. Samples were taken at the indicated time points and the abundance of ROCK1 and ERC1 was determined by western blotting. **D.** Expression of ROCK1 and ERC1 along infection with AD169 HCMV strain. Protein levels were analyzed by western blotting.

Since targeted degradation of central host proteins by the virus is not limited to immune ligands, we expanded our search and looked for anti-correlations between synthesis rate and steady state protein levels for all human genes for which we had both footprints and proteomic measurements [12]. We limited our search to the simplest profile in which both the footprints measurements and protein levels temporal profiles fit a linear regression (R2 > 0.8) and we focused on cases in which the footprints were upregulated (positive slope), whereas steady state protein levels were downregulated (negative slope). These criteria generated a list of 65 proteins that presented profiles that suggest active degradation (S7 Table, Figs 7A and S15A–S15C, left panels). When the same search was conducted with opposite criteria (requiring that the footprints will be downregulated and protein levels will be upregulated) we found no proteins that passed these conditions, supporting the notion that the list we obtained is biologically meaningful. We focused on five proteins from this list that reflect diverse biological functions; 1. ROCK1—a Rho-associated kinase that is a central regulator of actin cytoskeleton [51]. 2. ERC1—a regulatory subunit of the IκB kinase (IKK) complex [52]. 3. CDC37- a co-chaperone that binds to numerous kinases and promotes their interaction with the HSP90 complex [53]. 4. WDR61- a subunit of the human polymerase associated factor (PAF) and SKI complexes that regulate transcription[54]. 5. TIPRL- an inhibitory regulator of protein phosphatase-2A (PP2A). We conducted simultaneous real-time PCR and western blot analysis on cell extracts along HCMV infection and could confirm profiles that suggest active degradation of ROCK1 and ERC1 (Figs 7B and S16A). CDC37, WDR61 and TIPRL also presented profiles that support the premise they might be degraded, but their downregulation was less prominent (S15A–S15C Fig, right panels). To further establish the increased degradation of these proteins during HCMV infection, we examined their half-lives by cycloheximide (CHX) chase assays. As shown in Fig 7C, both ROCK1 and ERC1 show a long half-life in uninfected cells, whereas, in HCMV infected cells their half-lives are considerably decreased (Figs 7C and S16B). Similar results were obtained for CDC37, WDR61 and TIPRL (S15D Fig).

**Fig 8:**
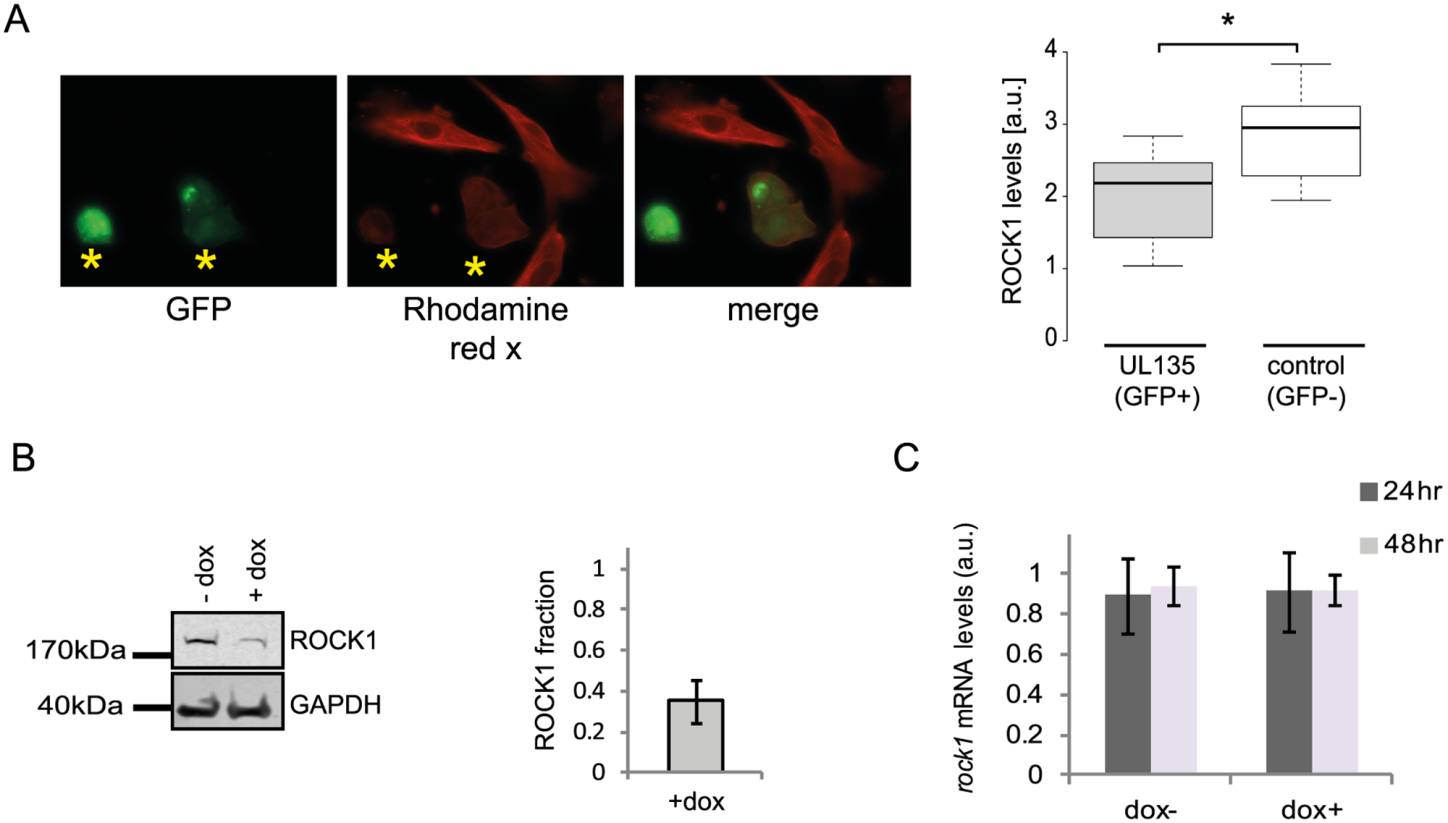
pUL135 contributes to ROCK1 degradation. **A.** Fluorescent microscopy images of cells expressing UL135 (GFP positive) and stained with ROCK1 antibody (rhodamine red X) (left panel). ROCK1 levels in 20 cells were quantified using Imaris (*p-val < 0.05) (right panel). **B.** HFF cells transduced with lentiviral vector expressing pUL135 under doxycycline inducible promotor were analyzed for ROCK1 expression levels, with or without the addition of doxycycline, using western blot analysis (left panel). Differences in protein levels were quantified using the Licor program (right panel). Error bar indicate the standard deviation in the ratio of ROCK1 expression in cells with and without doxycycline addition between two independent experiments. **C.** Measurements of *rock1* mRNA levels in HFF transduced with pUL135 under doxycycline inducible promoter, 24 and 48 hours after addition of doxycycline. Each experiment was performed in triplicates and average results are presented. Error bars indicate standard deviation of the mean.

We next tested if the proteins we identified are still degraded when cells are infected with a HCMV laboratory-adapted strain, AD169, in which a 15 kb composing the ULb’ region (genes UL133–UL150) is deleted. Importantly, ERC1, CDC37, WDR61 and TIPRL showed similar reduction in protein levels during infection with the AD169 virus (Figs 7D and S15E). However, ROCK1 levels were significantly elevated during AD169 infection (Fig 7D), suggesting that its degradation might depend on a protein/s that are encoded in the ULb’ region. ROCK1 plays a central role in actin regulation and its KD resulted in rounding up of cells (S17 Fig). pUL135 which is encoded in the ULb’ region was recently shown to remodel the actin cytoskeleton [55]. We therefore tested the effect of pUL135 on ROCK1 expression. We expressed pUL135 in fibroblasts using a lentiviral vector. The vector also contained GFP that was expressed from the same transcript using IRES and was used to identify cells that express the pUL135 protein. As a control, we expressed another viral protein pUL26 in the same manner. As was previously reported [55], expression of pUL135 in fibroblasts induced dramatic changes in cell morphology and cells became rounded up (Fig 8A, GFP expressing cells). We examined ROCK1 expression by immunofluorescence and observed a significant reduction in ROCK1 levels only in cells that express pUL135 (Figs 8A and S18). Similar reduction in ROCK1 levels following pUL135 expression was observed by immunoblotting (Fig 8B) and we confirmed that pUL135 expression did not affect ROCK1 mRNA levels (Fig 8C). Although pUL135 clearly affects ROCK1 expression the magnitude of the observed reduction strongly suggests that additional proteins contribute to ROCK1 degradation.

Overall, our results demonstrate that integration between dynamic translation and proteome measurements enables systematic identification of proteins that are targeted for degradation during infection. Since targeted degradation indicates a strong biological importance this approach could facilitate the discovery of central host functions during viral infection.

## Discussion

In this study, we present a comprehensive resource describing temporal changes in cellular gene transcription and translation along HCMV infection. During infection, HCMV extensively manipulates cellular gene expression to maintain conditions favorable for efficient viral propagation.

Analysis of transcription profiles has been the focus of systematic characterization of gene expression during infection [6,8,9,5,56]. However, control of protein production reflects both regulation of mRNA levels and the efficiency with which these messages are translated into proteins. Systematically measuring translation and mRNA levels allowed us to quantitatively evaluate their relative contributions and to reveal novel insights into the viral life cycle.

Our results show that the majority of cellular transcripts, changed by more than 3-fold along infection, exemplifying the radical changes that occur in cells during lytic HCMV infection. Our measurements show that this extensive gene regulation is dominated by changes in mRNA levels, but translational control also regulated the magnitude and timing of protein production during HCMV infection (discussed below).

Although the majority of the pathways that significantly changed during infection were previously mapped, our deep measurements and siRNA experiments allowed us to reveal that peroxisomes might be playing an unrecognized role during HCMV infection. Peroxisomes participate in central pathways of cellular metabolism such as β-oxidation of fatty acids (especially fatty acid chains that are too long to be handled by the mitochondria), amino acid catabolism and detoxification of reactive oxygen species [57]. It will be important to test which of these peroxisome functions are important for HCMV progression.

A more global look at the pathways that are elevated during infection reveals upregulation of modules that are important for cell proliferation (cell cycle, translation and transcription), whereas downregulated modules are related to development and cell-to-cell communication. Since an important dichotomy in the life of a cell is between proliferation and differentiation, these gene expression profiles illustrate that the cell program in infected cells is shifted, and although infected cells are not dividing, their cellular program resembles the cell program of a proliferating transformed cell.

Our simultaneous measurements of mRNA levels and translation rates along infection allowed us to quantitatively evaluate the role of translation regulation in controlling cellular genes expression and to expand recent findings [17]. Using stringent criteria we identified significant changes in TE for almost 10% of cellular genes. By clustering translationally regulated genes based on their TE values, we revealed several distinct temporal profiles suggesting that several independent molecular mechanisms are responsible for the observed translational changes. We were able to connect two of these clusters to known cellular modules; one cluster of genes was significantly enriched in mTORC1 targets implying that the increased translation of mRNAs from this cluster is attributed to mTORC1 activation by the virus [58]. This observation is also supported by the finding that ectopic expression of UL38 (mTORC1-activator) recapitulates a large fraction of the genes whose translation was stimulated by HCMV [17]. A second cluster we identified was enriched in genes involved in cell cycle progression and in mRNAs that showed enhanced translation at G1 and S phases of the cell cycle [40]. Since HCMV infection arrests the cells in a unique G1/S phase it is likely that the induced translation of these mRNAs is the consequence of cell cycle arrest. We observed additional translation regulation profiles, but these were not significantly enriched for any annotated biological process and additional work is needed to delineate the cellular mechanism that drives these changes and their relation to HCMV infection. Overall, our results suggest that functionally related groups of genes are translationally co-regulated and this provides an additional mean to control the expression of a particular subset of mRNAs. Transcription studies have enabled the identification of *cis*- and *trans*-transcriptional elements that control diverse cellular processes, whereas a similarly broad understanding of the importance and mechanisms of translational control remains much more elusive. Our data set and clustering approach provides a valuable basis for identifying such *cis*- and *trans*-translational regulators.

Unlike many viruses (including several Herpesviruses), HCMV does not completely suppress the synthesis of host proteins in infected cells [1,2]. Our approach allowed, for the first time, to test whether the virus manages to manipulate the cellular translation machinery to preferably translate its own mRNA. By globally comparing the TE of host genes to that of viral genes we made two novel observations; 1. When infection starts, viral mRNAs are translated less efficiently than host mRNAs. This observation intriguingly suggests that the cell possess intrinsic means to distinguish between cellular and viral mRNAs. 2. At 72 hpi a subtle but significant advantage for viral mRNAs translation is observed, suggesting that late in infection the virus does deploy the translation machinery to biasedly translate its mRNAs. Even though this virus does not fully co-opt the translation machinery like other viruses, elucidating the regulatory mechanisms underlying translational reprogramming of both the virus and the host can reveal novel modules the virus relies on, which could ultimately lead to the development of novel therapeutic strategies.

Our work also demonstrates that mass spectrometry and ribosome profiling represent highly complementary approaches; our comparison between changes in rate of protein synthesis, measured by ribosome profiling, and protein abundance, measured by mass spectrometry, revealed novel examples of regulated degradation of cellular proteins during HCMV infection. This integration of ribosome profiling with mass spectrometry measurements along a dynamic process, presents a novel unbiased approach to map protein degradation.

HCMV has evolved a variety of mechanisms to evade the immune response to survive in infected hosts, including elimination of cellular immune ligands from being presented on the cell surface. We reveal here that BTN2A1 and IGSF8, members of the Ig superfamily, are degraded during HCMV infection. BTN2A1 is a cell surface glycoprotein related to the extended family of B7 costimulatory molecules. It was shown to act as a ligand for DC-SIGN [59], a specific dendritic cell receptor, but its role in immune response was never investigated. IGSF8 was identified as a major tetraspanin (CD9 and CD81)-associated protein [60] and was shown to regulate the formation and maintenance of immune synapses [61]. It is therefore possible that degradation of IGSF8 allows protection against cytotoxic immune effector cells by interfering with immune synapse formation. A recent study revealed that US2, a viral protein that was originally defined by its capacity to target MHC molecules for degradation [44], is a pleotropic modulator of cell surface receptors [47]. Hsu *et al*. [47] unbiased measurements suggested that the US2 protein downregulates BTN2A1 and IGSF8 cell surface expression. Indeed, we show that BTN2A1 degradation is dependent on US2 expression but our analysis indicate that IGSF8 is still degraded even when US2 is not expressed. Our results suggest that US9 probably also plays a role in mediating IGSF8 degradation. Therefore, presenting an additional example for the redundancy in mechanisms HCMV is using to escape immune recognition. Interestingly, US9 was recently shown to selectively target MICA*008, a highly prevalent stress-induced ligand, to proteasomal degradation [49]. Our results suggest that like US2 [47], US9 probably targets a broader set of proteins and future work will have to address the specificity of this viral protein. More broadly, our ability to unbiasedly identify two immune ligands that are degraded during infection strongly argues for the validity of our approach.

Our analysis revealed additional cellular proteins that are degraded during infection. ROCK1 plays a critical role in mediating the effects of small GTPase RhoA on stress fiber formation, focal adhesion and cell motility [62]. Interestingly, these structures were shown to be modulated during HCMV infection [63,64] and the possible role of ROCK1 in modulation of these processes could be studied. We also show that ERC1 is degraded during HCMV infection. ERC1 was shown to be critical for NF-κB activation following ATM induction by genotoxic stress [65]. Since it is known that ATM is activated during HCMV infection [28,66], targeted degradation of ERC1 could serve as an elegant way to antagonize innate immunity response through attenuation of NF-κB signaling.

Finally, our approach is applicable to other viruses or any other pathogen and is useful to gain mechanistic insights into pathogen interference with regulation of mRNA expression, translation and protein degradation.

## Materials and Methods

### Cells and viruses

Human fibroblasts (CRL-1634) and the HCMV Merlin strain (VR-1590) were obtained from American Type Culture Collection (ATCC). The virus was propagated twice on HFF cells before the preparations of samples for sequencing. Cells were grown on 15cm plates and were infected at a multiplicity of infection (MOI) of 5.

The AD169, AD169VarL, AD169VarL-BAC, AD169VarL-BAC deltaUS9 and the AD169VarL-BAC deltaUL40 were previously described [49,67,68].

### Lentiviral constructs and stable cell lines

US2, US9 and UL135 were amplified from cDNA derived from HCMV-infected cells and cloned into the lentiviral vector pHAGE-DsRED(−)-ZsGreen(+). UL135 was cloned under the doxycycline inducible promoter.

Lentivirus was packaged by co-transfection of constructs with the 2nd generation packaging plasmids pMD2.G and PsPax using jetPEI (Polyplus-transfection) into 6-well plates with 293T cells according to protocol. 60 hours post transfection supernatants were collected and centrifuged at 1500 rpm for 5 minutes and filtered through a 0.45 μm filter. HFF expressing BTN2A1, IGSF8 or UL135 cell lines were generated by lentiviral transduction. 12 hours after infection fresh media was added, for UL135 cell line, the medium was supplemented with tet-free serum (Biological industries). Cells were analyzed for expression of proteins using fluorescent microscopy for GFP positive cells.

### Ribosome profiling and mRNA-Seq samples preparation

Cylcoheximide treatment was carried out as previously described [3]. Cells were lysed in lysis buffer (20mM Tris 7.5, 150mM NaCl, 5mM MgCl2, 1mM dithiothreitol, 8% glycerol) supplemented with 0.5% triton, 30 U/ml Turbo DNase (Ambion) and 100μg/ml cycloheximide, ribosome protected fragments were then generated as previously described [3]. Total RNA was isolated from infected cells using Tri-Reagent (Sigma).

Polyadenylated RNA was purified from total RNA sample using Oligotex mRNA mini kit (Qiagen). The resulting mRNA was modestly fragmented by partial hydrolysis in bicarbonate buffer so that the average size of fragments would be ~ 80bp. The fragmented mRNA was separated by denaturating PAGE and fragments 50–80 nt were selected as previously described [3]

### Sequence alignments, normalization and clustering

Prior to alignment, linker and polyA sequences were removed from the 3’ ends of reads. Bowtie v0.12.7 (*5*) (allowing up to 2 mismatches) was used to perform the alignments. First, reads that aligned to human rRNA sequences were discarded. All remaining reads were aligned to the concatenated viral (NC_006273.2) and human (hg19) genomes. Finally, still-unaligned reads were aligned to 200bp sequences that spanned splice junctions. Reads with unique alignments were used to compute the total number of reads at each position. Footprints and mRNA densities were calculated in units of reads per kilobase per million (RPKM) in order to normalize for gene length and total reads per sequencing run.

TE was calculated for genes that had more than uniquely aligned 150 reads of mRNA and footprints. For the comparison of between the virus and the host TE only genes with TE >1 were included. For clustering only genes with calculated expression > 3 RPKM in at least one of the condition and a change greater than 3-fold were used. Partitioning clustering was performed using Partek Genomic suits across mRNA, footprints, and TE data. Where indicated, gene lists were analyzed by Ingenuity Pathway Analysis (Ingenuity Systems, Redwood City, CA, USA) using default settings.

### 5’UTR characterizations

The 5′UTRs were obtained using the known gene ID from the UCSC Genome Browser (GRCh37/hg19). For Each Cluster the 5′UTR length, %G+C content and Gibbs free energy was calculated and compared to background list using Wilcoxon two-sided test.

The translationally regulated clusters we identified (Fig 3B) were compared to genes that were identified as transitionally upregulated during different phases of the cell cycle [40] and to genes that were translationally repressed after mTOR inhibition (log2 ratio <-1, Thoreen *et al*. [38]) and the enrichment was calculated using hyper Geometric test.

### Western blot analysis

Cells were lysed using RIPA buffer. Lysates were nutated at 4°C for 10 min, then centrifuged at 20,000 × g for 15 min at 4°C. Samples were then separated by 4–12% polyacrylamide Bis-tris gel electrophoesis (Invitrogen), blotted onto nitrocellulose membranes and immunoblotted with primary antibodies (αROCK1 ab134181; αERC1 ab180507; αCDC37 ab108305; αWDR61 ab57840; αB7H6 ab138588; αTIPRL ab70795; αGFP (ZsGreen) 632474 (Takara-Clontech); αIE1/IE2 (CH160) ab53495 (Abcam); αHA 3F10 (Roche); αGAPDH 2118S (Cell signaling); αATG3 A3231 (Sigma); αUL44 (CMV ICP36) CA006 (Virusys); αpp28 CA004 (Eastcoast). Secondary antibodies used were Goat anti-rabbit, Goat anti-mouse (IRDye 800CW or IRDye 680RD, Licor), or Goat anti Rat (Alexa Fluor 680, ab175778, Abcam). Reactive bands were detected by Odyssey CLx infrared imaging system (Licor). Protein concentration was measured by Bradford assay (Sigma cat no. B6916). Protein quantification was performed on Licor software.

### Real-time PCR

Total RNA was extracted using Tri-Reagent (Sigma) according to protocol. cDNA was prepared using High-Capacity cDNA Reverse Transcription Kit (ABI) according to protocol.

Real time PCR was performed using the SYBR Green PCR master-mix (ABI) on a real-time PCR system StepOnePlus (life technologies) with the following primers (forward, reverse):

RAB12; GCCGTCATGGAAGGTTATTT, CCCTTAGGAAGCCATGAGAG

IRAK1; CAGACAGGGAAGGGAAACAT, AATCACTGTGAAGCCTGTGC

HSP90AB1; GCAGACATCTCCATGATTGG, AAGGAACCTCCAGCAGAAGA

SIX5; CAGTCACCACATCCTTCTGC, GGGAGGGCTGTAACAGAGAG

FAT1; CATCATTGTTGCCAAACCTC, GAGGACGATGGTCATTTGTG

FAT4; AGTGGTGGAACCTGTCACAA, CTCTGCAGGCACTCATTGAT

LAMA2; GGCCTGACTGGGAAATTAAA, CTCGGAAATTCCACAAACCT

ATP5J: GGTGTTACAGCAGTGGCATT, CCTCTCCAGCTCTTGCTGAT

NCOA6; GTCCTGGGTCCAGTAGGAAA, GAGGAGTGGGACTGACCAAT

TSEN54; CCAAGACCTGCCACTGTCTA, GGACAGAGCTTGGTTGGAAT

POLR2L; AGGAGAGCCTTCCATCTCG, ATCTGGCTCTTCAGATTCCG

CDC37; GGTAAATACCAAGCCCGAGA, ATGCCAAAGTGCTTGATCTG

B7H6; ACCCTGGGACTGTCTACCAG, TGAAATAGGCCACCAATGAA

ERC1; TGCAAATCAGAAAGCTGACC, TGGTGGTAGAGGTGGTC

TIPRL; TCCCTGAAATGATGTTTGGA, CTTCAGCACAGGCCACTTTA

ROCK1; TTGGTAGAGGTGCATTTGGA, AAAGCCATGATGTCCCTTTC

WDR61; TGCTCATATTCGTCTTTGGG, ACTTTCCCGACATGAGTTCC

MFGE8; CACTCTGCGCTTTGAGCTAC, TCCAGCTGAAGAGATGCAAG

IGSF8; ACCCTATTTGTGCCTCTGCT, ACAGTCGACACCTGCAAGAC

BTN2A1; AGAGGAATCCACAGGACCAC, GGGACTTAGCCACCCTTACA

RAB12; GCCGTCATGGAAGGTTATTT, CCCTTAGGAAGCCATGAGAG

BTN2A1; AGAGGAATCCACAGGACCAC, GGGACTTAGCCACCCTTACA

### Knockdowns

Cells were transfected with siRNA validated for each of the target genes or negative control (TriFECTa Kit DsiRNA Duplex, IDT) in the presence of Lipofectamine RNAiMAX reagent (Life Technologies), according to manufacturer's standard protocol. 24 hours after transfection, cells were infected with HCMV (Merlin strain, MOI 3). All experiments were performed in triplicate, and representative results are reported.

### TCID_50_ assay

10^4^ HFF were plated in 96-well plates and cells were infected with 10-fold serial dilutions of supernatant from knocked-down infected cells, collected 5 days post infection. At 10 days post infection the dilutions showing cytopathic effect were evaluated by light microscopy. The TCID_50_/ml was calculated using the Spearman-Kaerber method [69]. Experiments were performed at least 3 times and representative figure is presented.

### Degron based live cell translation reporter

The fluorescence-based translation reporter was cloned using fusion pcr of three parts; 1. DHFR(Y100I) 2. sfGFP-NLS-P2A 3. NLS-mCherry and cloned into the pHR lentiviral expression vector using BstXI-NotI. 5’ UTRs were inserted using BstX1-BsiWI. Primers to amplify the UTRs used in this study were based on the RNA-seq data to represent the most common UTR splice variant in HFF cells.

### Time-lapse microscopy and quantification of fluorescence

All live cell-imaging experiments were performed at 37°C on a AxioObserver Z1 widefield microscope using a 20x air objective and Axiocam 506 mono camera. Cells were grown and imaged in 24-well glass bottom plates and 1 hr before imaging normal growth medium was replaced with DMEM without phenol red, supplemented with 10% FCS and antibiotics. Image analysis was done in Imaris software. For image quantification, images were first corrected for background subtraction using default settings. Segmentation and tracking of each field was performed on the mCherry channel and the GFP mean intensity over time for each segment was measured. The average GFP slope for all segments for each sample was calculated (10<n).

### Immunofluorescence

Cells were plated on ibidi slides and fixed in 4% paraformaldehyde for 30 min, washed in PBS (pH 7.4) and permeabilized with 0.2% Triton X-100 in PBS for 10 min, then blocked with 3% BSA in PBS for 30 min. Detection of ROCK1 was performed by immunostaining with anti-ROCK1 antibodies (abcam 156284, 1:200 in PBS) 1hr, RT. Cells were washed 3 times with PBS and labeled with anti-rabbit Rhodamine Red-X-conjugated secondary antibody (Jackson ImmunoResearch 711-295-152, 1:200 in PBS) 1 hr, RT. Imaging was performed on a AxioObserver Z1 widefield microscope using a 63x oil objective and Axiocam 506 mono camera.

### Mass spectrometry analysis of IGSF8-interacting proteins

Samples were digested by trypsin, analyzed by LC-MS/MS on Q Exactive (Thermo). The data was analyzed with Protein Discoverer 1.4 versus Human and HCMV Uniprot database and against decoy databases (in order to determine the false discovery rate -FDR), using the Sequest search engine. The data was also analyzed vs the specific sequences of HCMV Merlin strain. Identifications were filtered with high identification confidence refers to 0.01 FDR, top rank, mass accuracy, and a minimum of 2 identified peptides in the human proteins. Semi-quantitation was done by calculating the peak area of each peptide. The area of the protein is the average of the three most intense peptides from each protein.

### Pull down

HFF stably expressing IGSF8-HA or empty vector were collected from 2x15 cm tissue culture plates 48 hours after mock infection or infection with HCMV Merlin strain at MOI ~ 5. 12 hours before harvesting, the proteosomal inhibitor MG-132 was added in final concentration of 10μM. Cells were washed twice in PBS and lysed in 1 ml lysis buffer (150mM NaCl, 2mM CaCl_2_, 2mM MgCl_2_, 1% NP-40 in PBS, supplemented with Roche complete protease inhibitor cocktail). Lysis was facilitated by nutating the cells 1 hr at 4°C. Cells were then centrifuged for 15 min, 20,000 rpm 4°C and supernatant was separated and incubated with pre-equilibrated anti-HA magnetic beads (Pierce). Tagged protein binding to beads was performed by nutating cells-beads mixture at 4°C for 1 hr.

Tagged protein bound to anti-HA magnetic beads was separated using magnetic stand, beads were washed 3 times in wash buffer (same composition as lysis buffer with 0.1% NP-40). Elution was performed by incubating the beads with 0.1M glycine pH 2.5 with gentle mixing. Eluate was neutralized with 0.1M Tris pH 8.5. Protein sample buffer was added to eluates and samples were resolved on Bis-Tris-SDS gel 4–12% and stained with Instant Blue staining. The gel was sent to Mass-spectrometry analysis for identification of interacting proteins.

## Supporting Information

S1 Fig Reproducibility of the ribosome occupancies and mRNA measurements of host genes at 5hpi. The correlations in footprints and mRNA measurements between biological replicates are presented.
(TIF)

S2 Fig **(A)** Patterns of average RNA-expression and ribosome profiling data of each of the clusters from Fig 2A. (* is added for p-val < 0.05). **(B)** Real-time PCR and western blot analysis for representative mRNAs or protein from different clusters. In real-time PCR level of gene expression was normalized by the amount of *polr2l* or *mfge8* mRNA. Each experiment was performed in triplicates. Western blot analysis was performed on total cell lysates.
(TIF)

S3 Fig The “Role of BRCA1 in DNA Damage Response” pathway based on Ingenuity. Individual proteins (circles) are shaded if they are part of cluster 1.
(TIF)

S4 Fig The “DNA Mismatch Repair signaling” pathway based on Ingenuity. Proteins (circles) are shaded if they are part of cluster 1.
(TIF)

S5 Fig The “eIF2 signaling” pathway based on Ingenuity. Individual proteins (circles) are shaded if they are part of cluster 2.
(TIF)

S6 Fig The “Assembly of RNA Polymerase II” pathway based on Ingenuity. Individual proteins (circles) are shaded if they are part of cluster 2.
(TIF)

S7 Fig The “Protein Ubiquitination Pathway” pathway based on Ingenuity. Individual proteins (circles) are shaded if they are part of cluster 5.
(TIF)

S8 Fig The “Hepatic Fibrosis” pathway based on Ingenuity. Individual proteins (circles) are shaded if they are part of clusters 3 and 8.
(TIF)

S9 Fig The “Metalloproteinase Inhibition” pathway based on Ingenuity. Individual proteins (circles) are shaded if they are part of cluster 3 and 8.
(TIF)

S10 Fig **(A)** FACS analysis of propidium iodide cell viability assay performed on siRNA transfected cells, 48 hours post transfection. In each panel, right side represents percentage of live cells and left side represents the percentage of dead cells. **(B)** Measurements of mRNA levels of indicated genes after transfection with siRNAs or scrambled non-targeting siRNA. HFF cells were transfected with siRNAs and after 24hr either mock-infected or infected with the Merlin strain. 48 hours post infection RNA samples were collected and analyzed by real time PCR. For each gene, 2 siRNA were tested. Each real-time PCR experiment was performed in triplicates and average results are presented. **(C**) Cells transfected with a control or a second siRNA targeting various host genes were infected with the Merlin strain (MOI = 3). After 5 days **s**upernatants were collected and viral titers were calculated by TCID50. **(D)** Differences in protein levels presented in Fig 2C were quantified using the Licor program. Reference expression level was set as expression of proteins in the cells transfected with negative control siRNA.
(TIF)

S11 Fig **(A)** TE was calculated by dividing RPKM of ribosome footprints with RPKM of mRNA measurements and the correlation in TE of host genes between biological replicates is represented. **(B)** 5'UTR %G+C content and **(C)** 5'UTR length boxplots for genes in clusters presented in Fig 3B(light purple) and background (all known 5’UTRs, gray). All clusters were compared to background by the Wilcoxon two-sided test. (* is added for p-val < 0.05). **(D)** Quantification of images from 10 cells (mean and SD) with or without cycloheximide addition to block protein synthesis.
(TIF)

S12 Fig Biological replicates for time points 5 and 72 hours post HCMV infection showing differences in TE between viral and human genes. (TIF)

S13 Fig Ribosome profiling and RNA-seq data of known and putative immune ligands, compared with temporal protein expression [12]. (TIF)

S14 Fig Differences in protein levels of BTN2A1 **(A)** and IGSF8 **(B)** along HCMV infection with different strains, relative the GFP expression from the same plasmid, were quantified using the Licor program. (TIF)

S15 Fig **(A-C)** Ribosome profiling data and temporal protein expression [12] for genes whose protein product seems to be degraded during HCMV infection, compared with real-time PCR and western blot analysis. Real-time PCR data was normalized by the amount of *mfge8* mRNA. Each experiment was performed in triplicates. Western blot analysis was performed on cell lysates and GAPDH was used as loading control. **(D)** Cells were mock-infected or infected with HCMV for 48hr and cycloheximide was added to the medium to stop protein translation. Samples were taken at the indicated time points and the abundance of CDC37, WDR61 and TIPRL was determined by western blotting. **(E)** Expression of CDC37, WDR61 and TIPRL along infection with AD169 HCMV strain. Protein levels were analyzed by immunoblotting and GAPDH was used as loading control.
(TIF)

S16 Fig Differences in protein levels of ROCK1 and ERC1 along HCMV infection **(A)** and after treatment with CHX **(B)**, were quantified using the Licor program. (TIF)

S17 Fig Fluorescent microscopy images of HFF transfected with siRNA for *rock1* knockdown versus wt cells using phalloidin staining. (TIF)

S18 Fig Cells expressing either pUL135 or pUL26 (that was used as a control) together with GFP were stained with ROCK1 antibody (rhodamine red X). ROCK1 levels were quantified from 20 cells using Imaris (* p-val < 0.05).
(TIF)

S1 Table RPKM of human genes RNA levels (mRNA) and translation (footprints) along HCMV infection. (XLSX)

S2 Table Full enrichment analysis for the 10 clusters presented in Fig 2A using the Database for Annotation, Visualization and Integrated Discovery, DAVID [20]. Each sheet presents the analysis of the indicated cluster.
(XLSX)

S3 Table Full pathway enrichment analysis for the 10 clusters presented in Fig 2A using Ingenuity. Each sheet presents the analysis of the indicated cluster.
(XLSX)

S4 Table Full upstream regulator analysis for the 10 clusters presented in Fig 2A using Ingenuity. Each sheet presents the analysis of the indicated cluster.
(XLSX)

S5 Table Fold change in TE compared to the mock sample for human genes for which significant changes in TE were observed. (XLSX)

S6 Table Enrichment analysis for the 5 clusters presented in Fig 3B, using Ingenuity. Each sheet presents the analysis of the indicated cluster. (XLSX)

S7 Table Human genes that present expression profiles suggesting degradation during HCMV infection. The third to ninth columns present the protein abundance measurements along HCMV infection taken from [12]. The tenth, eleventh and twelve columns give the slope, Y Intercept and R^2^ values of the calculated linear regression for protein expression, respectively. The thirteenth to seventeenth columns present the normalized footprint densities along infection. The eighteenth to twentieth columns give the slope, Y Intercept and R^2^ values (respectively) of the calculated linear regression for translation at the first 4 time points.
(XLSX)
