# Supplementary figures and images for: The Transcription and Translation Landscapes during Human Cytomegalovirus Infection Reveal Novel Host-Pathogen Interactions
Source: PLoS Pathog. 2015 Nov 24;11(11):e1005288. doi: 10.1371/journal.ppat.1005288 (PMC4658056; doi:10.1371/journal.ppat.1005288)

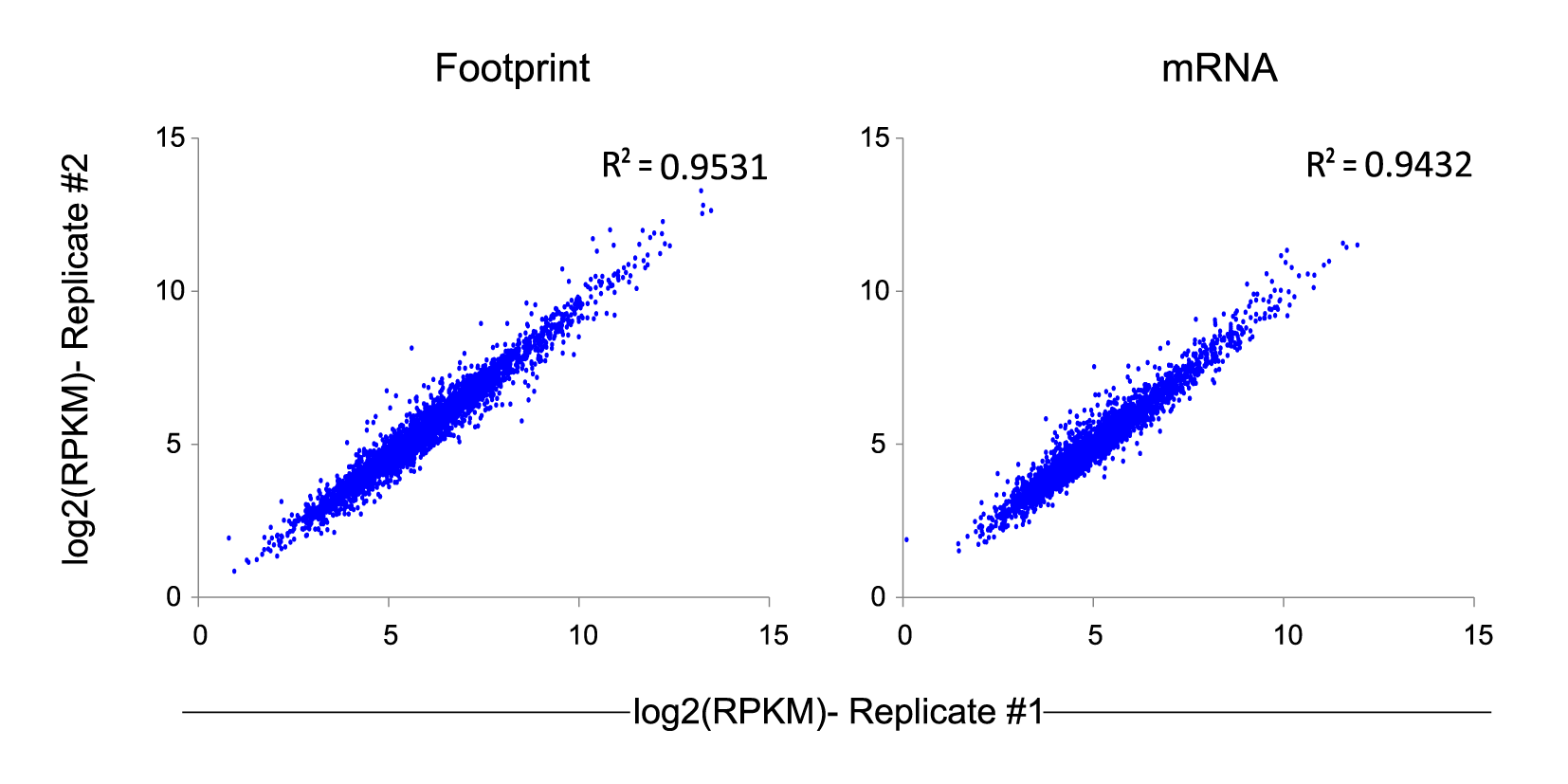

Supplement: S1 Fig — The correlations in footprints and mRNA measurements between biological replicates are presented. (TIF) [file ppat.1005288.s001.tif]

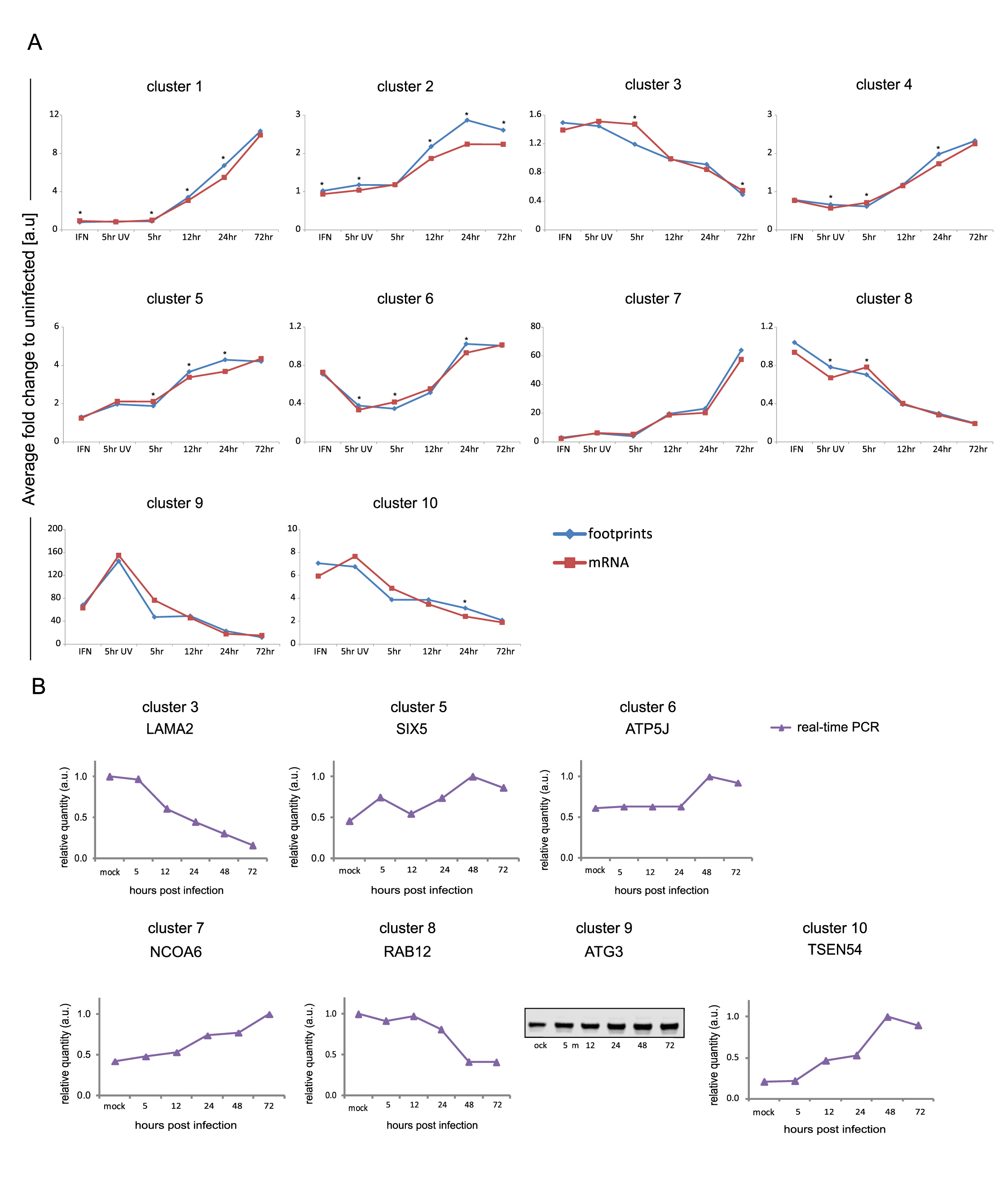

Supplement: S2 Fig — (A) Patterns of average RNA-expression and ribosome profiling data of each of the clusters from Fig 2A. (* is added for p-val < 0.05). (B) Real-time PCR and western blot analysis for representative mRNAs or protein from different clusters. In real-time PCR level of gene expression was normalized by the amount of polr2l or mfge8 mRNA. Each experiment was performed in triplicates. Western blot analysis was performed on total cell lysates. (TIF) [file ppat.1005288.s002.tif]

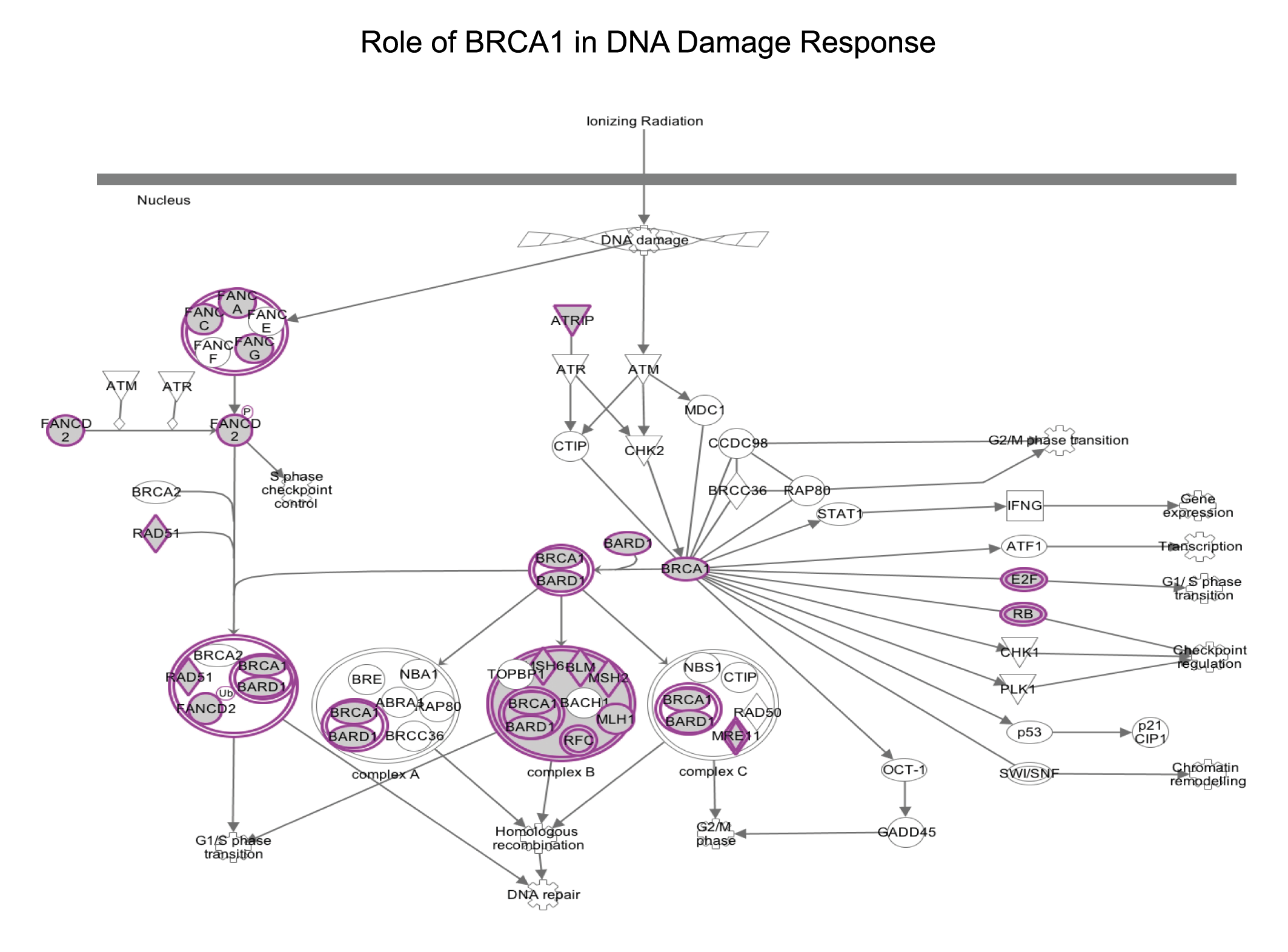

Supplement: S3 Fig — Individual proteins (circles) are shaded if they are part of cluster 1. (TIF) [file ppat.1005288.s003.tif]

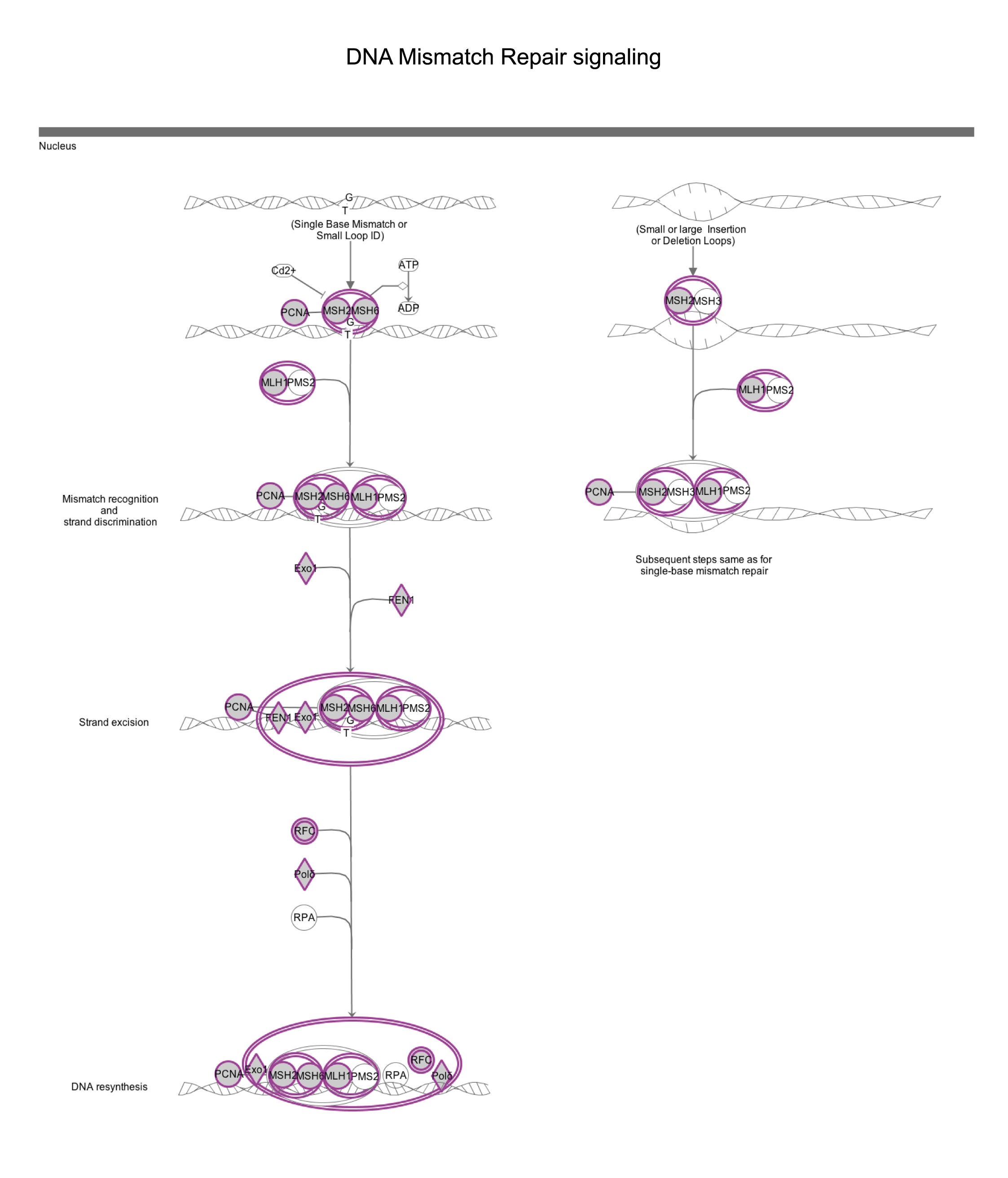

Supplement: S4 Fig — Proteins (circles) are shaded if they are part of cluster 1. (TIF) [file ppat.1005288.s004.tif]

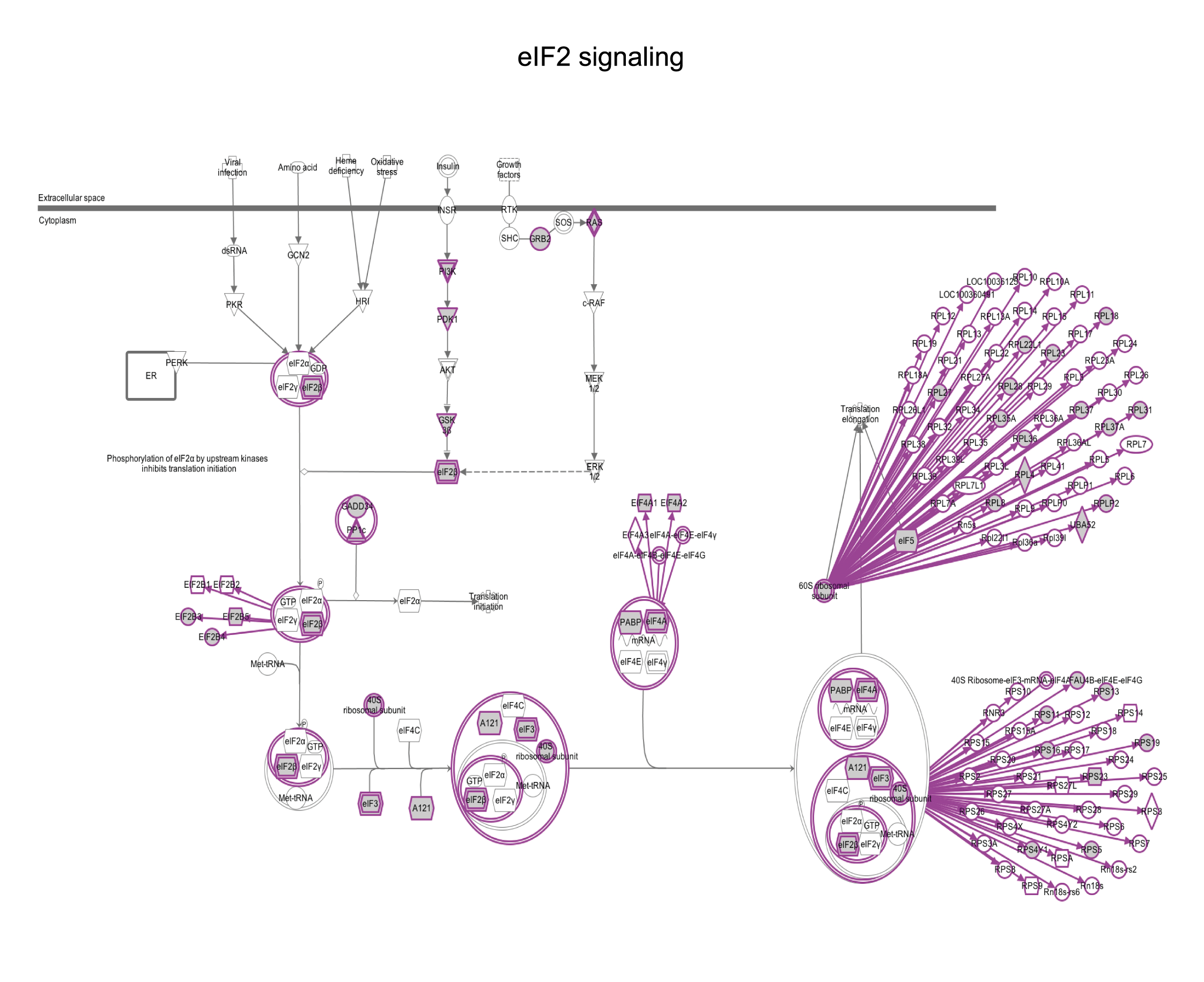

Supplement: S5 Fig — Individual proteins (circles) are shaded if they are part of cluster 2. (TIF) [file ppat.1005288.s005.tif]

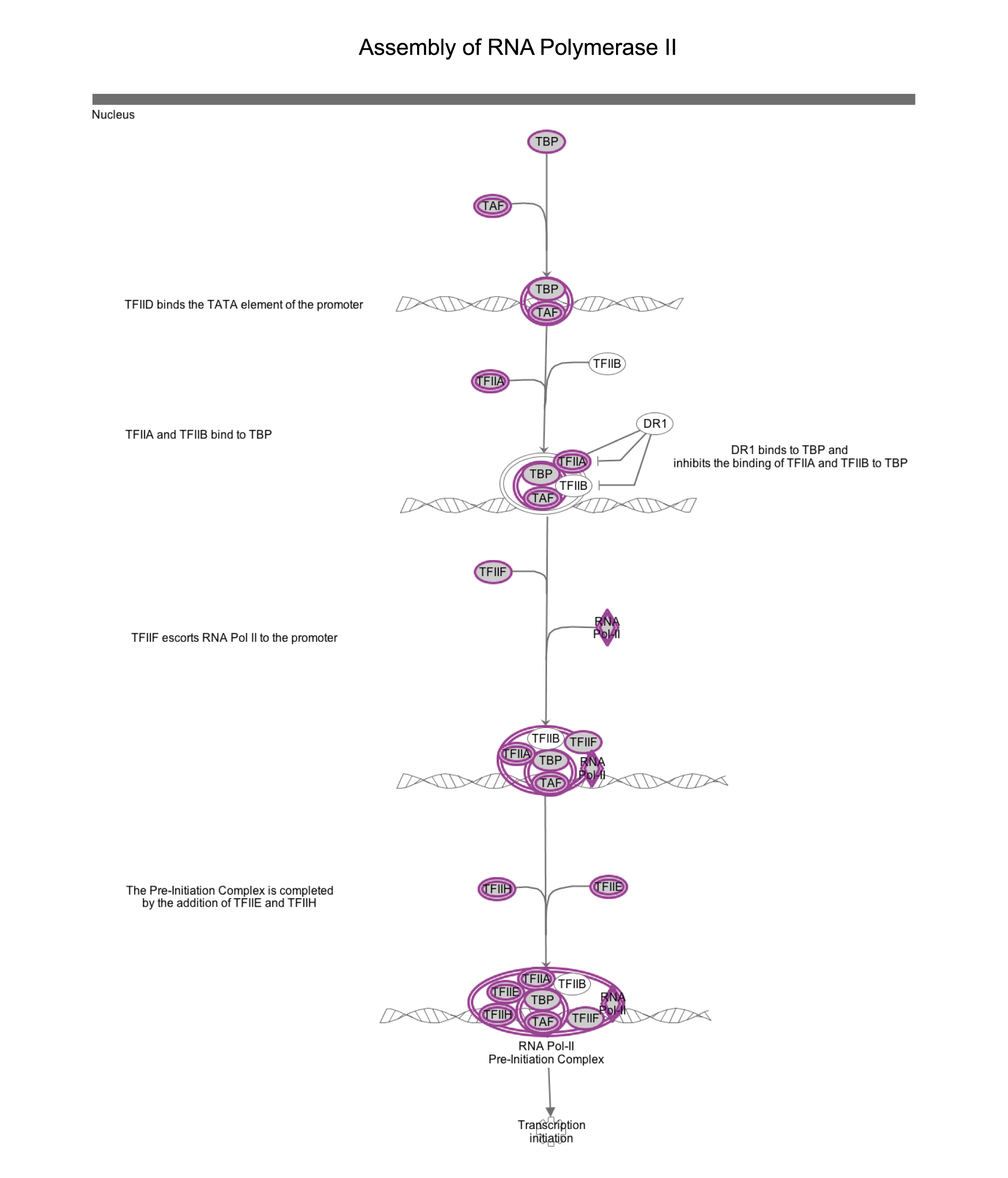

Supplement: S6 Fig — Individual proteins (circles) are shaded if they are part of cluster 2. (TIF) [file ppat.1005288.s006.tif]

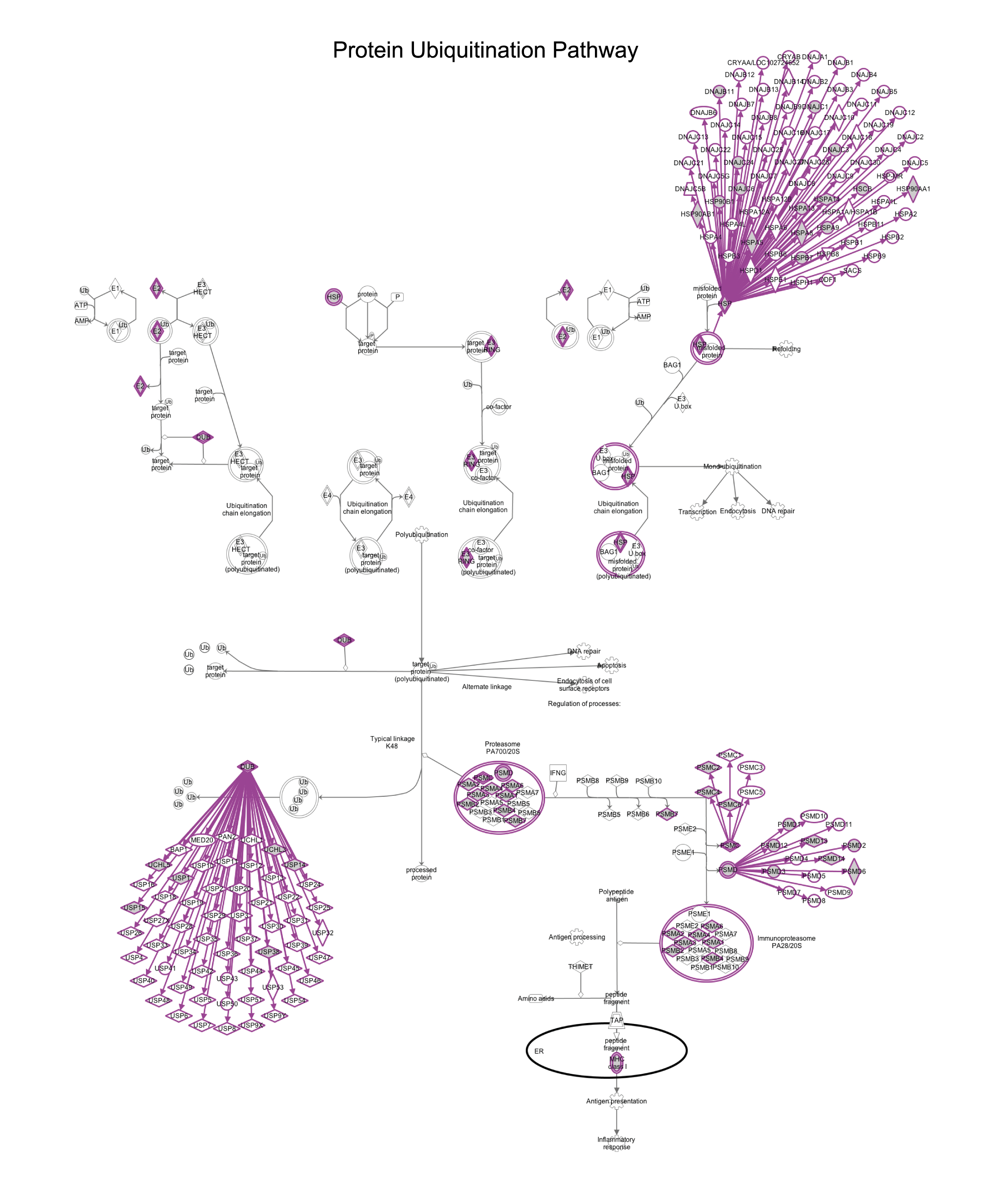

Supplement: S7 Fig — Individual proteins (circles) are shaded if they are part of cluster 5. (TIF) [file ppat.1005288.s007.tif]

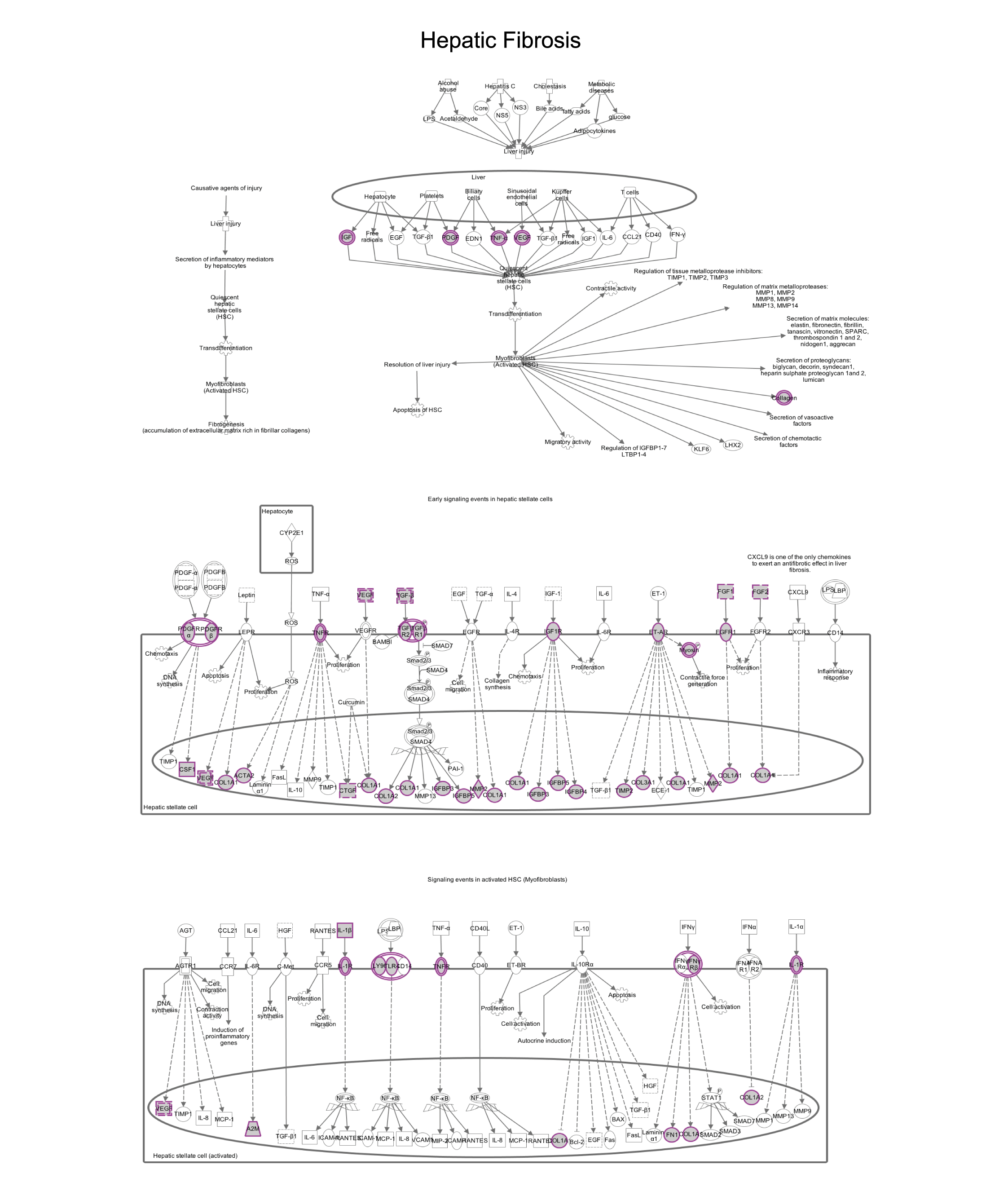

Supplement: S8 Fig — Individual proteins (circles) are shaded if they are part of clusters 3 and 8. (TIF) [file ppat.1005288.s008.tif]

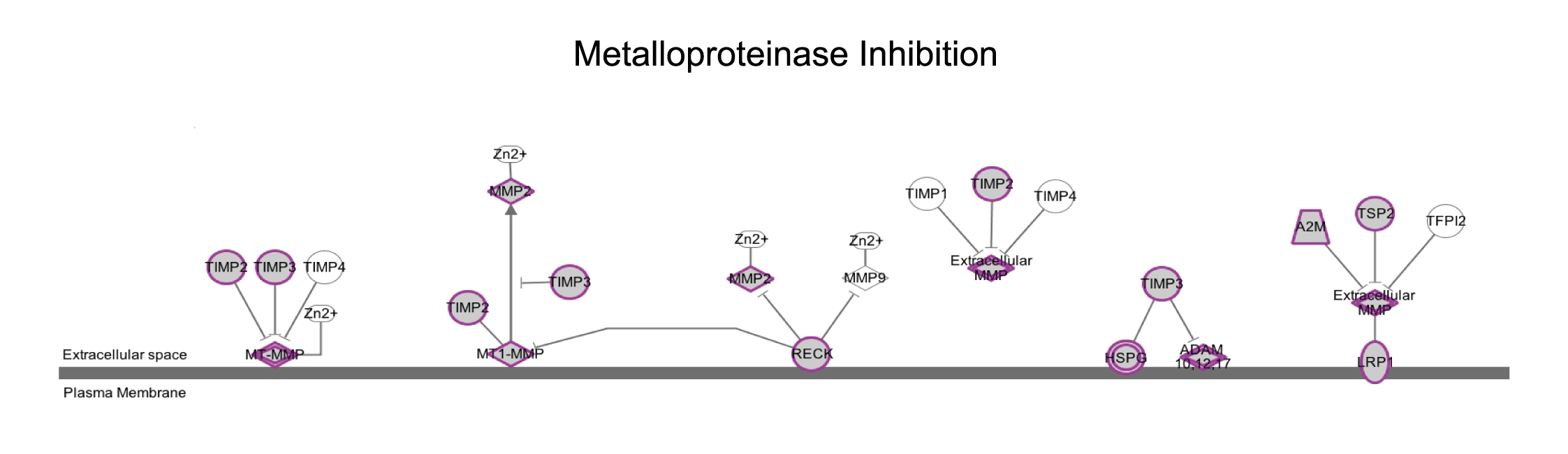

Supplement: S9 Fig — Individual proteins (circles) are shaded if they are part of cluster 3 and 8. (TIF) [file ppat.1005288.s009.tif]

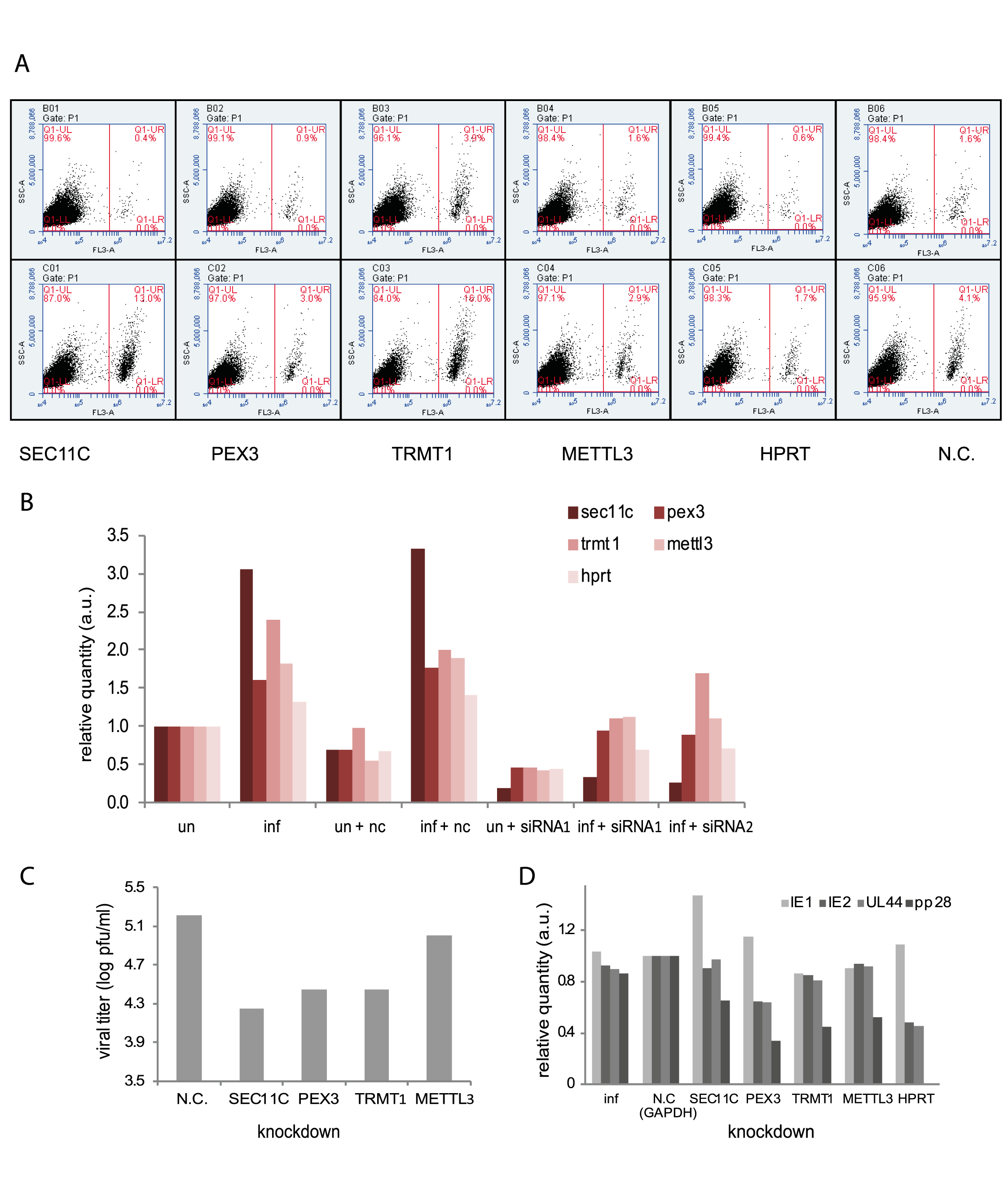

Supplement: S10 Fig — (A) FACS analysis of propidium iodide cell viability assay performed on siRNA transfected cells, 48 hours post transfection. In each panel, right side represents percentage of live cells and left side represents the percentage of dead cells. (B) Measurements of mRNA levels of indicated genes after transfection with siRNAs or scrambled non-targeting siRNA. HFF cells were transfected with siRNAs and after 24hr either mock-infected or infected with the Merlin strain. 48 hours post infection RNA samples were collected and analyzed by real time PCR. For each gene, 2 siRNA were tested. Each real-time PCR experiment was performed in triplicates and average results are presented. (C) Cells transfected with a control or a second siRNA targeting various host genes were infected with the Merlin strain (MOI = 3). After 5 days supernatants were collected and viral titers were calculated by TCID50. (D) Differences in protein levels presented in Fig 2C were quantified using the Licor program. Reference expression level was set as expression of proteins in the cells transfected with negative control siRNA. (TIF) [file ppat.1005288.s010.tif]

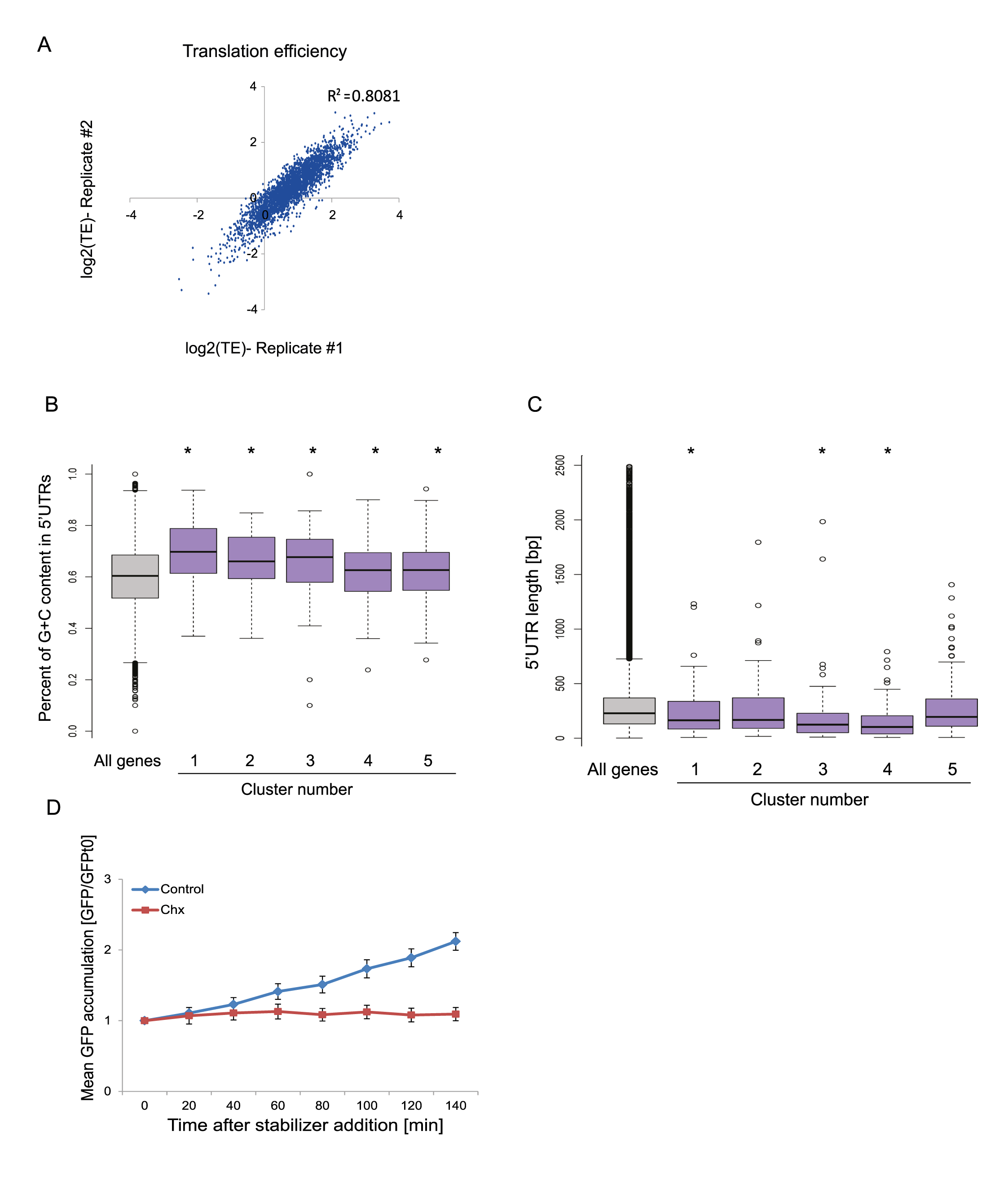

Supplement: S11 Fig — (A) TE was calculated by dividing RPKM of ribosome footprints with RPKM of mRNA measurements and the correlation in TE of host genes between biological replicates is represented. (B) 5'UTR %G+C content and (C) 5'UTR length boxplots for genes in clusters presented in Fig 3B(light purple) and background (all known 5’UTRs, gray). All clusters were compared to background by the Wilcoxon two-sided test. (* is added for p-val < 0.05). (D) Quantification of images from 10 cells (mean and SD) with or without cycloheximide addition to block protein synthesis. (TIF) [file ppat.1005288.s011.tif]

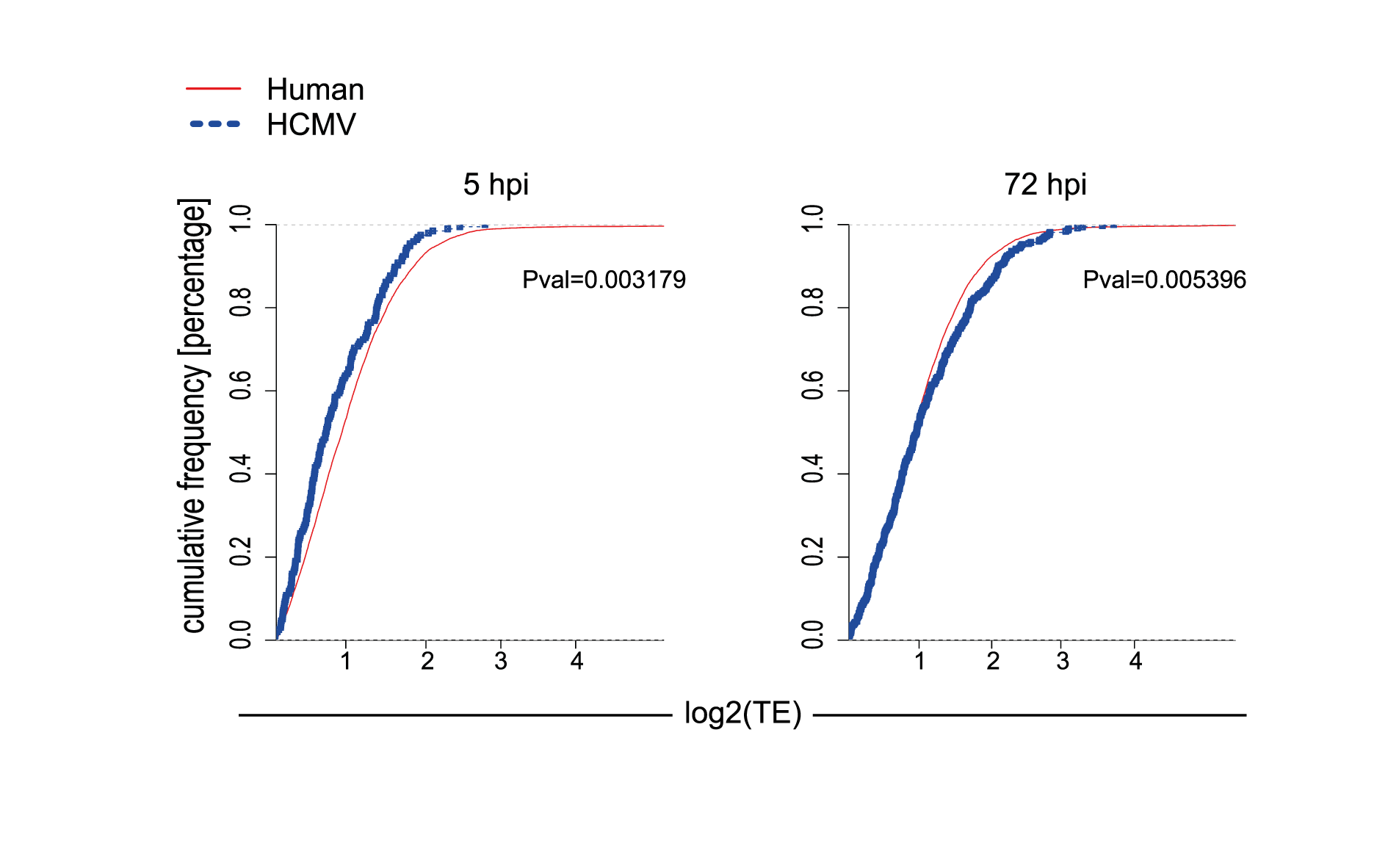

Supplement: S12 Fig — (TIF) [file ppat.1005288.s012.tif]

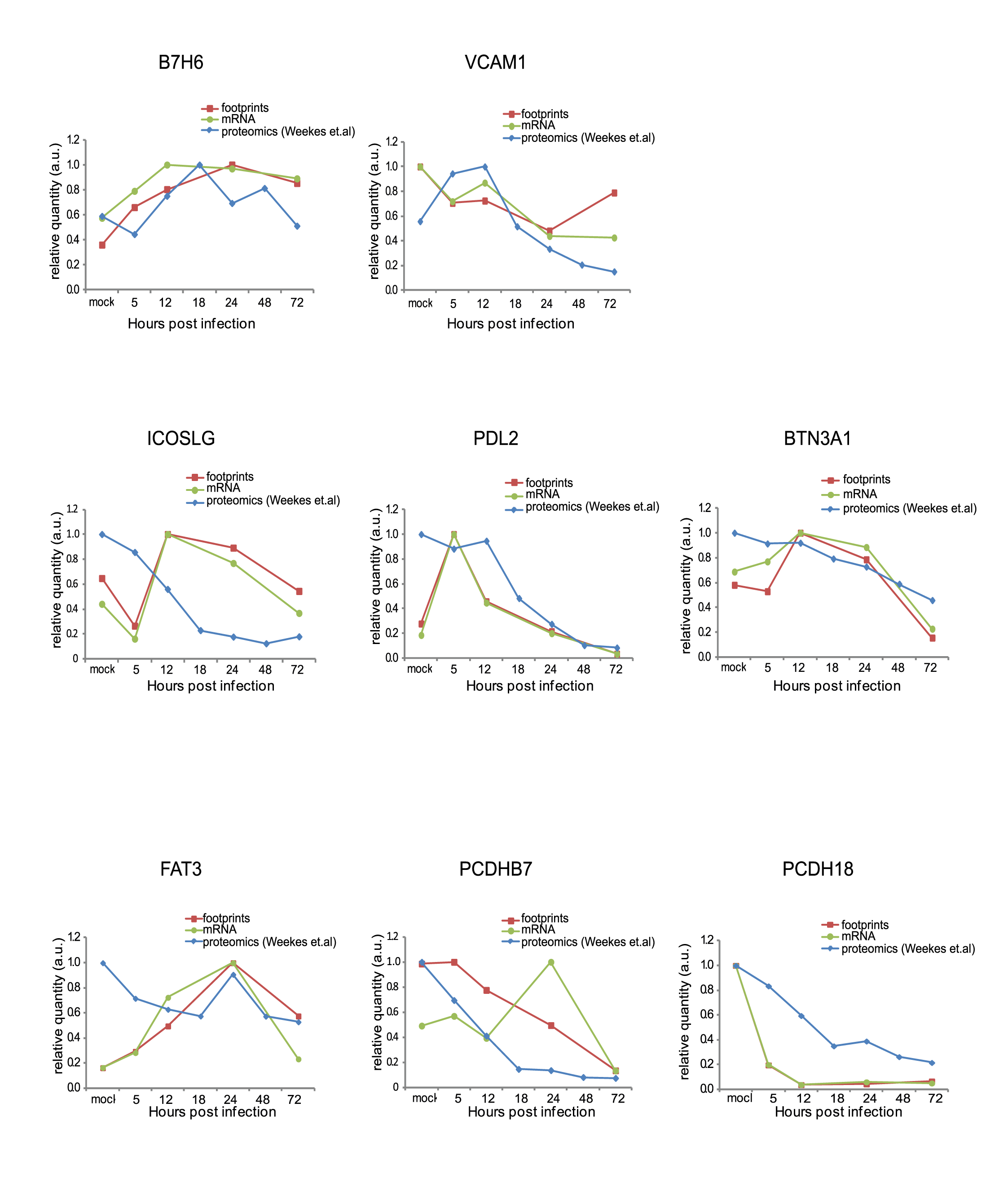

Supplement: S13 Fig — (TIF) [file ppat.1005288.s013.tif]

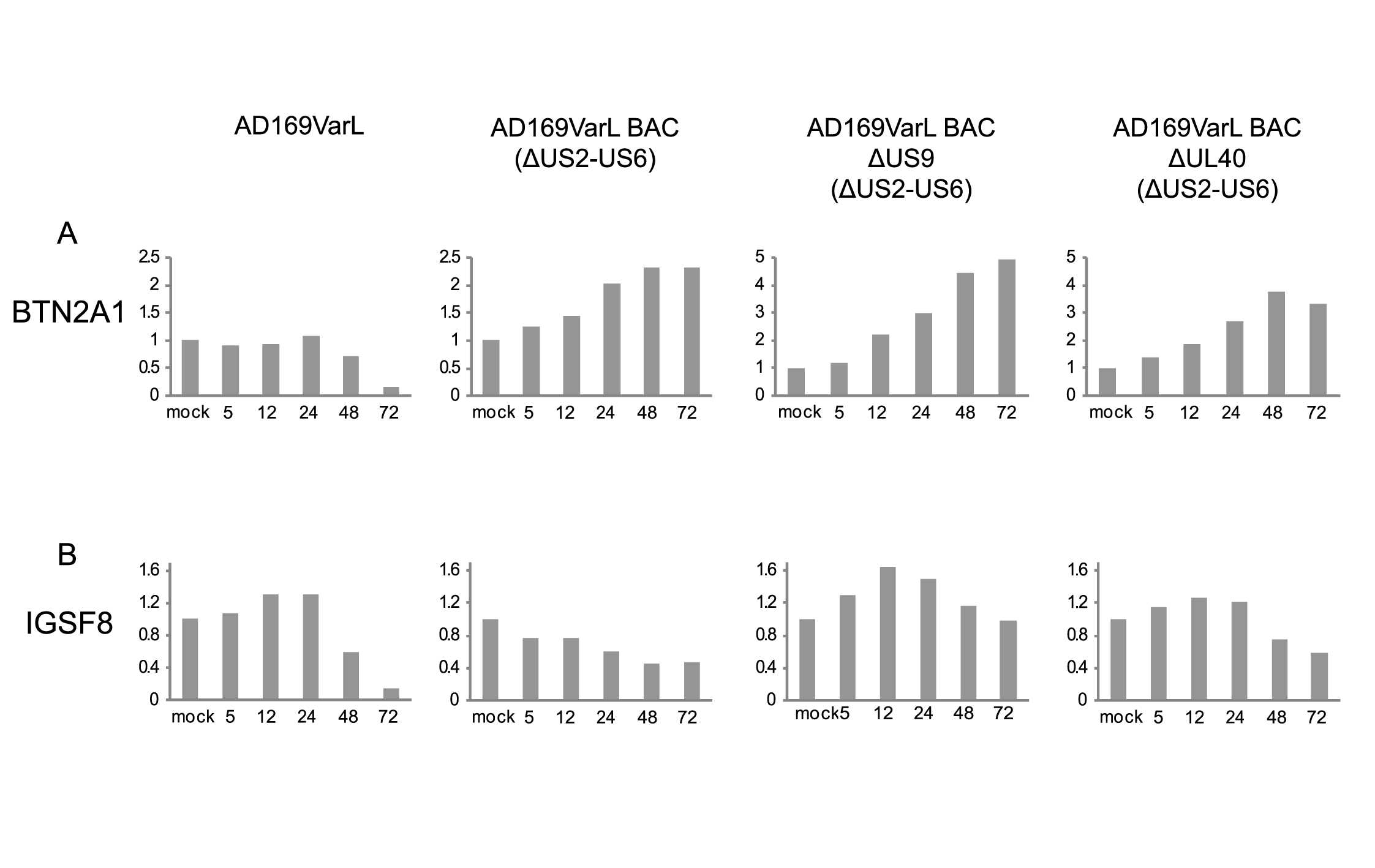

Supplement: S14 Fig — (TIF) [file ppat.1005288.s014.tif]

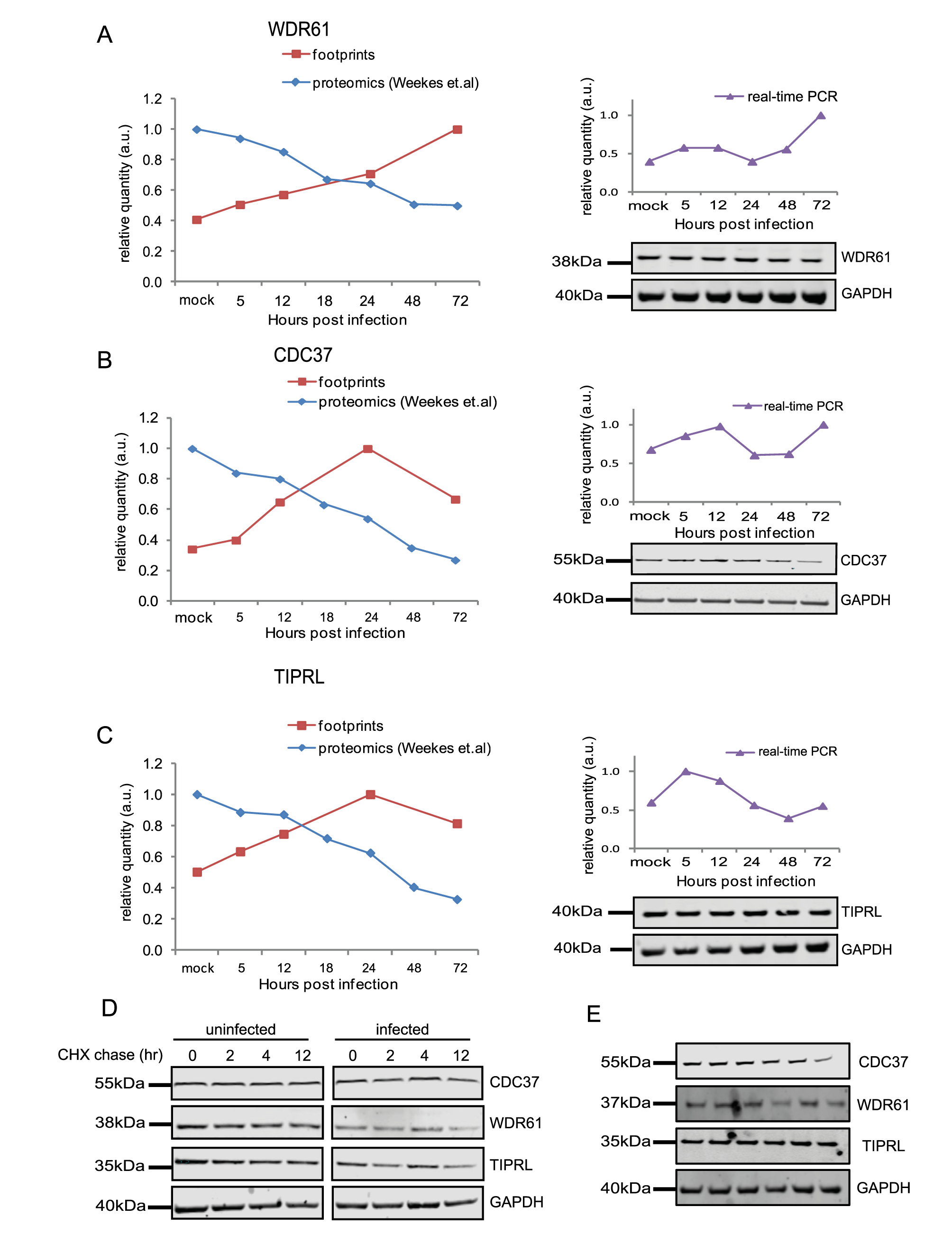

Supplement: S15 Fig — (A-C) Ribosome profiling data and temporal protein expression [12] for genes whose protein product seems to be degraded during HCMV infection, compared with real-time PCR and western blot analysis. Real-time PCR data was normalized by the amount of mfge8 mRNA. Each experiment was performed in triplicates. Western blot analysis was performed on cell lysates and GAPDH was used as loading control. (D) Cells were mock-infected or infected with HCMV for 48hr and cycloheximide was added to the medium to stop protein translation. Samples were taken at the indicated time points and the abundance of CDC37, WDR61 and TIPRL was determined by western blotting. (E) Expression of CDC37, WDR61 and TIPRL along infection with AD169 HCMV strain. Protein levels were analyzed by immunoblotting and GAPDH was used as loading control. (TIF) [file ppat.1005288.s015.tif]

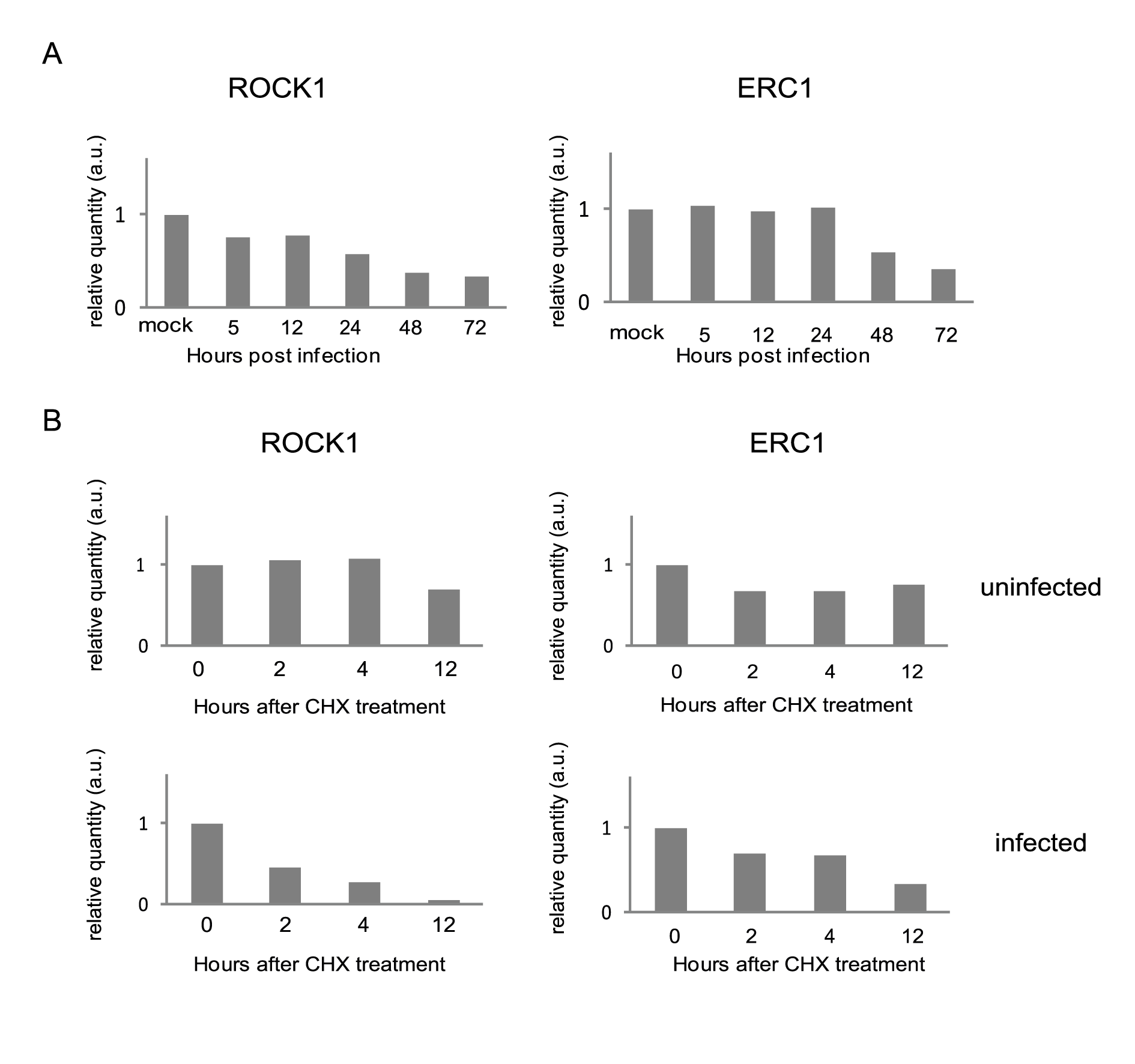

Supplement: S16 Fig — (TIF) [file ppat.1005288.s016.tif]

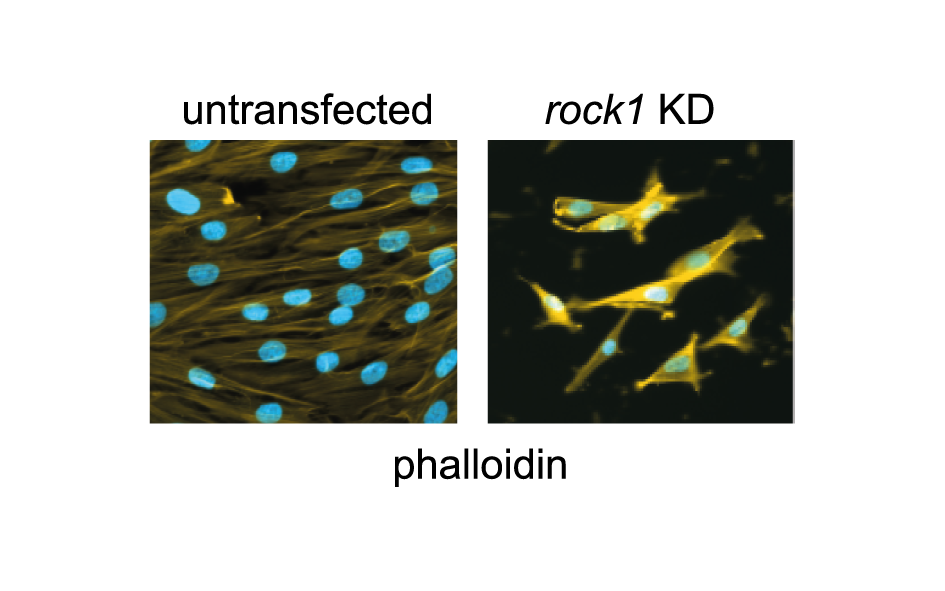

Supplement: S17 Fig — (TIF) [file ppat.1005288.s017.tif]

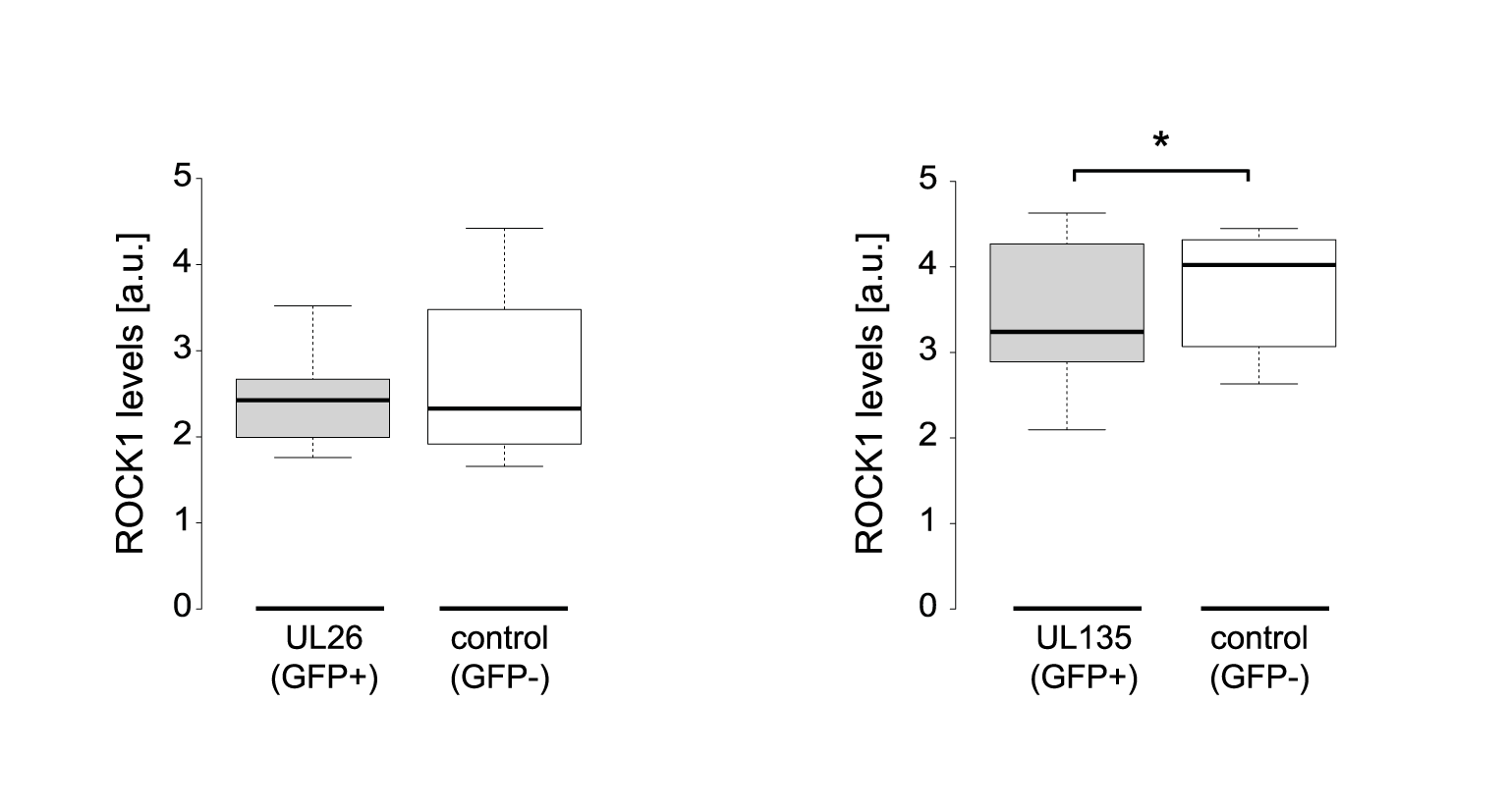

Supplement: S18 Fig — ROCK1 levels were quantified from 20 cells using Imaris (* p-val < 0.05). (TIF) [file ppat.1005288.s018.tif]
